# Dual-atom pairs with electron buffering enable ultrastable-cycling all-solid-state lithium–sulfur batteries

**DOI:** 10.1093/nsr/nwag024

**Published:** 2026-01-15

**Authors:** Jiwei Shi, Mingyang Jiang, Chuannan Geng, Zhonghao Hu, Yun Cao, Jiaqi Lan, Li Wang, Quan-Hong Yang, Wei Lv

**Affiliations:** Shenzhen All-Solid-State Lithium Battery Electrolyte Engineering Research Center, Key Laboratory of Electrocatalytic Materials and Green Hydrogen Technology of Guangdong Higher Education Institutes, Shenzhen Key Laboratory for Graphene-based Materials, Institute of Materials Research, Tsinghua Shenzhen International Graduate School, Tsinghua University, Shenzhen 518055, China; Shenzhen All-Solid-State Lithium Battery Electrolyte Engineering Research Center, Key Laboratory of Electrocatalytic Materials and Green Hydrogen Technology of Guangdong Higher Education Institutes, Shenzhen Key Laboratory for Graphene-based Materials, Institute of Materials Research, Tsinghua Shenzhen International Graduate School, Tsinghua University, Shenzhen 518055, China; Shenzhen All-Solid-State Lithium Battery Electrolyte Engineering Research Center, Key Laboratory of Electrocatalytic Materials and Green Hydrogen Technology of Guangdong Higher Education Institutes, Shenzhen Key Laboratory for Graphene-based Materials, Institute of Materials Research, Tsinghua Shenzhen International Graduate School, Tsinghua University, Shenzhen 518055, China; Shenzhen All-Solid-State Lithium Battery Electrolyte Engineering Research Center, Key Laboratory of Electrocatalytic Materials and Green Hydrogen Technology of Guangdong Higher Education Institutes, Shenzhen Key Laboratory for Graphene-based Materials, Institute of Materials Research, Tsinghua Shenzhen International Graduate School, Tsinghua University, Shenzhen 518055, China; Shenzhen All-Solid-State Lithium Battery Electrolyte Engineering Research Center, Key Laboratory of Electrocatalytic Materials and Green Hydrogen Technology of Guangdong Higher Education Institutes, Shenzhen Key Laboratory for Graphene-based Materials, Institute of Materials Research, Tsinghua Shenzhen International Graduate School, Tsinghua University, Shenzhen 518055, China; Shenzhen All-Solid-State Lithium Battery Electrolyte Engineering Research Center, Key Laboratory of Electrocatalytic Materials and Green Hydrogen Technology of Guangdong Higher Education Institutes, Shenzhen Key Laboratory for Graphene-based Materials, Institute of Materials Research, Tsinghua Shenzhen International Graduate School, Tsinghua University, Shenzhen 518055, China; Nanoyang Group, Tianjin Key Laboratory of Advanced Carbon and Electrochemical Energy Storage, State Key Laboratory of Chemical Engineering and Low-Carbon Technology, School of Chemical Engineering and Technology, National Industry-Education Platform for Energy Storage, and Collaborative Innovation Center of Chemical Science and Engineering, Tianjin University, Tianjin 300072, China; Haihe Laboratory of Sustainable Chemical Transformations, Tianjin 300192, China; Nanoyang Group, Tianjin Key Laboratory of Advanced Carbon and Electrochemical Energy Storage, State Key Laboratory of Chemical Engineering and Low-Carbon Technology, School of Chemical Engineering and Technology, National Industry-Education Platform for Energy Storage, and Collaborative Innovation Center of Chemical Science and Engineering, Tianjin University, Tianjin 300072, China; Haihe Laboratory of Sustainable Chemical Transformations, Tianjin 300192, China; Shenzhen All-Solid-State Lithium Battery Electrolyte Engineering Research Center, Key Laboratory of Electrocatalytic Materials and Green Hydrogen Technology of Guangdong Higher Education Institutes, Shenzhen Key Laboratory for Graphene-based Materials, Institute of Materials Research, Tsinghua Shenzhen International Graduate School, Tsinghua University, Shenzhen 518055, China

**Keywords:** all-solid-state lithium–sulfur batteries, dual-metal sites, buffered-atom catalysts, electron transfer, solid–solid interface

## Abstract

All-solid-state lithium–sulfur batteries (ASSLSBs) show promise in balancing high energy density and safety, but face the bottleneck of sluggish sulfur conversion kinetics. Single-atom catalysts (SACs) could alleviate this by maximizing active-site utilization and intimate contact with sulfur. However, the metal centers often undergo irreversible electronic reconstruction, causing fast degradation and capacity fading. Here, we report an atomic-scale electron buffering strategy with electronegativity-matched dual-metal atom sites, which strengthen interfacial bonding with sulfur species while maintaining stability. The dual-metal Cu and Ni atoms are anchored on the polymeric carbon nitride (Cu_1_Ni_1_–PCN) to form spatial proximity (∼3.4 Å) single-atom pairs with similar electronegativity. This pair mediates electron buffering between the metals, enabling dynamic valence modulation that suppresses deactivation and enhances interfacial *d–p* orbital hybridization with sulfur. Consequently, the catalyst enables consistently low activation energy during long cycling, retaining a high capacity of 948 mAh g^−1^ after 2500 cycles at 1 mA cm^−2^ and exhibiting almost no capacity decay over 7000 cycles at 2 mA cm^−2^. This work establishes electron buffering as a robust approach to stabilize atomic-scale catalysts and offers a general design framework for high-performance sulfur electrocatalysis in ASSLSBs.

## Teaser

Pairing copper and nickel atoms creates a self-regulating electron buffer that stabilizes reactive sites in all-solid-state lithiumsulfur batteries, enabling ultrastable cycling performance.

## INTRODUCTION

High-energy and reliable batteries are crucial for advancing sustainable energy solutions and modern technologies. Among emerging candidates, all-solid-state lithium–sulfur batteries (ASSLSBs) have attracted considerable attention due to their intrinsic safety, long-term durability and exceptionally high theoretical energy density of 2600 Wh kg^−1^, which far surpasses that of conventional lithium-ion batteries and positions them as a transformative next-generation technology [1–4]. However, the practical deployment of ASSLSBs remains challenging. The fundamental bottleneck lies in the intrinsically sluggish redox kinetics of sulfur and Li_2_S, originating from their electrical and ionic insulation. This limitation leads to poor sulfur utilization, high polarization and rapid capacity fading during long-term cycling [5,6]. Catalysis has been highlighted as a pivotal strategy for improving sulfur conversion kinetics in lithium–sulfur batteries, which has also been successfully extended to ASSLSBs in recent work [7]. However, the solid–solid conversion process lacks the liquid electrolyte-mediated mass transport, which restricts the effective contact between sulfur species and catalytic active sites, significantly constraining the solid–solid sulfur conversion efficiency [8].

**Figure 1.**
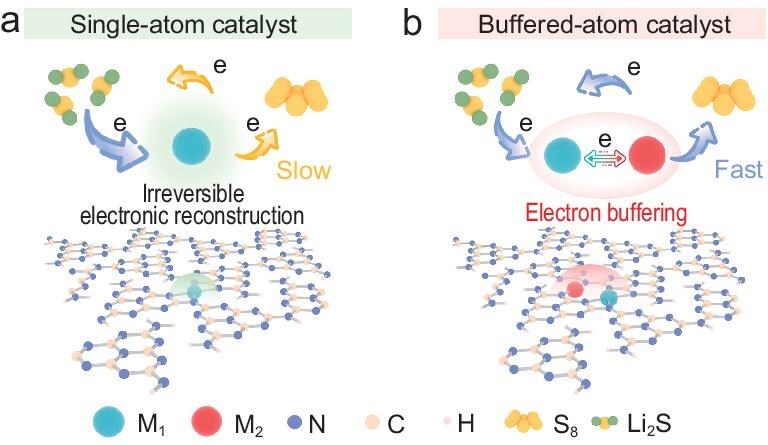
Schematic for sulfur species conversion facilitated by the BAC. In SAC (a), maintaining high activity of the single-metal atom center remains challenging due to irreversible electronic reconstruction at the solid–solid interface during the long-term cycling. However, with the introduction of the BAC (b), reversible electron buffering between adjacent metal sites induces dynamic valence modulation and *d*-band center reconstruction, which strengthen the *d–p* orbital hybridization with sulfur species, thereby enhancing the interfacial electronic interaction while keeping the stability.

Among the various catalysts, single-atom catalysts (SACs) with atomically dispersed active sites enable the sufficient contact of active sites with sulfur species at the solid–solid interfaces, showing promise to enhance the sulfur conversion efficiency [7]. However, in ASSLSBs, isolated metal centers undergo continuous electron transfer with sulfur species in the absence of liquid electrolyte-mediated mass transport, which drives irreversible electronic reconstruction of the active sites and leads to rapid catalytic deactivation during cycling. Thus, the question of how to achieve high activity and keep long-term stability at the same time challenges the use of SACs in ASSLSBs (Fig. 1a). Introducing an additional metal center to form dual active sites presents a promising strategy for fine-tuning of the *d*-band center, thereby optimizing interactions with intermediates and enhancing electron transfer [9–11]. In particular, this also enables balanced activity and stability due to electronic coupling between the two metal centers in heteronuclear dual single-atom catalysts (DACs) [12–14]. This coupling induces charge redistribution, facilitates the delocalization of accumulated charges during redox processes, and mitigates site-specific over-reduction or over-oxidation [15–19]. This provides a way to bring atomic-scale catalysts into practical use in ASSLSBs.

Herein, distinct from previously reported atomic-scale catalysts that rely on static electronic modulation of isolated metal sites, we design the dual-atom pair with the metal atoms having similar electronegativity, which achieves dynamic electron migration to generate an electron buffering effect. Such an effect enables stabilization of metal valence states and enhances *d–p* orbital hybridization with sulfur species (Fig. 1b). The concept is exemplified by anchoring Cu–Ni atomic pairs on polymeric carbon nitride (Cu_1_Ni_1_–PCN), in which Cu atoms can transfer electrons to the more electronegative Ni atoms. The PCN host, enriched with nitrogen coordination sites, defines a close interatomic distance of ∼3.4 Å between metal centers and ensures adaptive coordination during reactions [11]. During solid-state sulfur conversion, this intermetallic electron transfer shifts the *d*-band centers of the metal sites closer to the Fermi level, enhancing orbital hybridization with sulfur species and strengthening interfacial electronic interactions. This creates sufficient active sites at the constrained solid–solid interface and maintains their electronic integrity, effectively preventing irreversible site reconstruction. As a result, Cu_1_Ni_1_–PCN maintains a consistently low activation energy (*E_a_*) before and after cycling, enabling stable battery operation for over 7000 cycles at a current density of 2 mA cm^−2^ without capacity decay, far surpassing most reported ASSLSBs. This electron buffering principle provides a general pathway for stabilizing atomic-scale catalysts and advancing high-performance ASSLSBs.

## RESULTS AND DISCUSSION

### Catalyst synthesis and characterization

**Figure 2.**
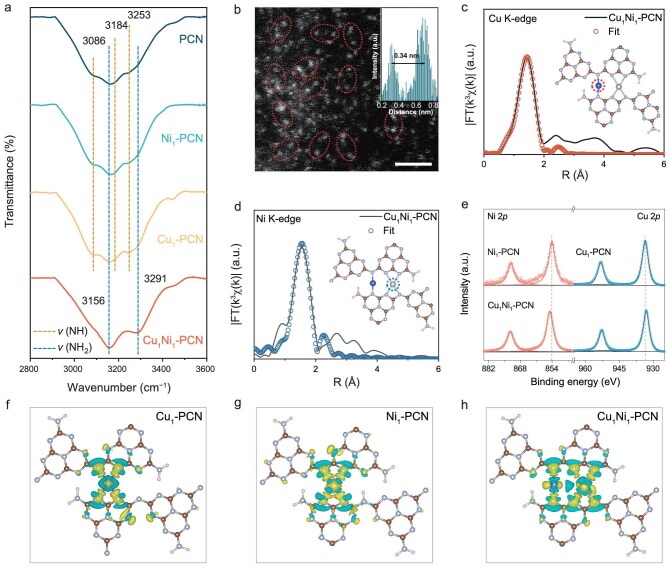
Characterization of the BAC. (a) FTIR spectra of different catalysts. (b) Atomic-resolution ADF-STEM image (buffered-atom pairs are circled). Scale bar, 1 nm. *k*^3^-weighted Cu (c) and Ni (d) K-edge Fourier-transformed (FT) EXAFS spectra of Cu_1_Ni_1_-PCN. (e) Cu 2*p* and Ni 2*p* XPS spectra of Cu_1_–PCN, Ni_1_–PCN and Cu_1_Ni_1_–PCN. Charge density differences for Cu_1_–PCN (f), Ni_1_–PCN (g) and Cu_1_Ni_1_–PCN (h). The yellow and cyan areas depict charge accumulation and depletion, respectively.

The rich and periodic N–H functional groups in PCN provide a well-defined coordination matrix for stabilizing isolated metal atoms [20,21]. Cu and Ni single atoms replace the H atoms to form Cu_1_–PCN, Ni_1_–PCN and Cu_1_Ni_1_–PCN, as evidenced by the almost complete reduction in the intensity of the N–H-related vibrational band in the Fourier transform infrared (FTIR) spectra of different catalysts (Fig. 2a and Table S1), and are further stabilized by the opposite N atoms [11]. The atomic dispersion of metal species was directly visualized by annular dark-field scanning transmission electron microscopy (ADF-STEM; Fig. 2b). In the case of Cu_1_Ni_1_–PCN (7.92 wt% Cu, 7.55 wt% Ni), bright spots corresponding to individual Cu and Ni atoms are homogeneously distributed, with a significant fraction arranged in close spatial proximity to form well-defined pairs. The average Cu–Ni distance is approximately 0.34 nm, determined by ADF-STEM and structural optimization, which is sufficiently short to enable support-mediated electronic coupling and reversible electron redistribution between the two metal centers. Powder X-ray diffraction (XRD) measurements further exclude the presence of nanoparticles or clusters (Fig. S1). Consistently, the extended X-ray absorption fine structure (EXAFS) spectra (Fig. 2c and d; Figs S2–S7 and Table S2) show prominent features centered at ∼1.5 Å and a weaker peak at ∼2.2 Å, corresponding to the first-shell Cu/Ni–N and second-shell Cu/Ni–C atomic distances, respectively (Fig. S2). X-ray absorption near-edge structure (XANES) analysis further reveals that the Cu K-edge absorption edge of the buffered-atom catalyst (BAC) lies between that of Cu foil and CuO (Fig. S3b), indicating a Cu valence state between 0 and +2. A similar trend is observed for the Ni K-edge (Fig. S3d), suggesting a Ni valence state also between 0 and +2. X-ray photoelectron spectroscopy (XPS) supports this finding and further confirms the electronic transfer between Cu and Ni atoms in the BAC. Compared to Cu_1_–PCN and Ni_1_–PCN, the Ni 2*p* and Cu 2*p* spectra of Cu_1_Ni_1_–PCN exhibit shifts toward higher and lower binding energies, respectively, indicating electron transfer from Cu to Ni atoms (Fig. 2e) [22,23]. The wavelet transform (WT) analysis of the Cu and Ni K-edge EXAFS spectra further excludes contributions from metal clusters or oxides (Figs S6 and S7). To gain further theoretical understanding, first-principles density functional theory (DFT) calculations were performed to compare the charge density differences among Cu_1_–PCN, Ni_1_–PCN and Cu_1_Ni_1_–PCN (Fig. 2f–h). The results reveal the presence of negative charge accumulation between Cu and Ni atoms in Cu_1_Ni_1_–PCN [14], demonstrating electron transfer and redistribution within the BAC system. Such interatomic electron delocalization not only confirms the strong synergistic interaction between Cu and Ni but also highlights the dynamic electron buffering capacity of the BAC, which plays a pivotal role in stabilizing the local electronic environment under redox conditions.

### High activity and stability of BAC

**Figure 3.**
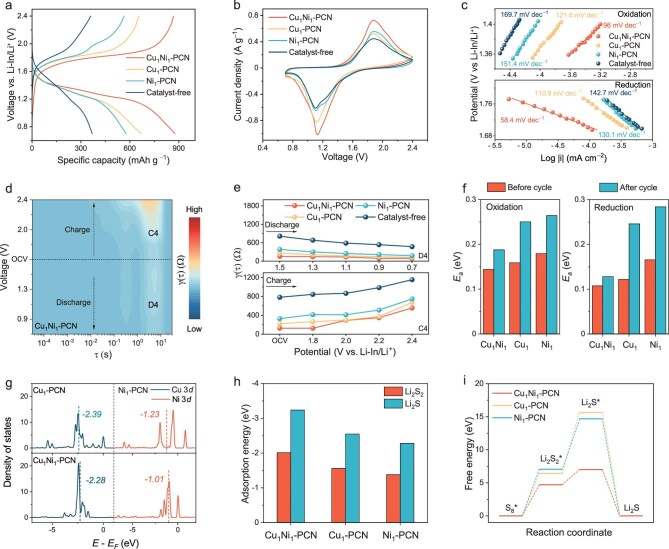
Kinetic studies of ASSLSBs with different catalysts. (a) Galvanostatic discharge–charge profiles at 0.1 mA cm^−2^ in the second cycle in the presence of different catalysts. (b) CV profiles of different cathodes at 25°C with a scan rate of 0.1 mV s^−1^. (c) Tafel slopes for cells with different cathodes. (d) The DRT curves plotted calculated from the *in situ* EIS results and the corresponding 2D intensity mapping of cathodes with Cu_1_Ni_1_–PCN during the charge and discharge process. (e) Evolution of relaxation-based function γ (τ) of the charge and discharge process for cathodes with different catalysts and without any catalyst. (f) Activation energies of the discharge and charge processes for different cathodes calculated from the CV profiles. (g) DOS for the 3*d*-orbitals of Cu and Ni for different catalysts and their *d*-orbital center positions. (h) The adsorption energies of Li_2_S_2_, and Li_2_S with different catalysts. (i) Gibbs free energy of sulfur reduction process on Cu_1_–PCN, Ni_1_–PCN and Cu_1_Ni_1_–PCN.

To demonstrate the high activity and stability of Cu_1_Ni_1_–PCN, we assembled ASSLSBs using a cathode composed of Ketjenblack, Li_2_S, catalysts and Li_6_PS_5_Cl (Figs S8–S11). The charge–discharge profiles of the battery with Cu_1_Ni_1_–PCN exhibit the lowest overpotential and the highest discharge capacity compared to those without a catalyst or with the Cu_1_-PCN/Ni_1_-PCN catalyst (Fig. 3a). At 0.1 mV s^−1^, the cyclic voltammetry (CV; Fig. 3b) curves also display a higher reduction peak potential (1.11 V) and a greater current density (0.94 A g^−1^). Furthermore, within the selected Tafel slope region (oxidation: 1.35–1.40 V; reduction: 1.69–1.77 V), the Cu_1_Ni_1_–PCN-based cathode demonstrates a lower Tafel slope (Fig. 3c), indicating enhanced electrochemical activity and improved redox kinetics [24]. The galvanostatic intermittent titration technique (GITT) was conducted to evaluate the reaction kinetics. All four samples exhibit characteristic discharge plateaus, and the Cu_1_Ni_1_-PCN-based cathode shows a higher specific capacity (Fig. S12a). As shown in Fig. S12b and S12c, the overpotential and Li^+^ diffusion coefficient (*D*_Li_^+^) at various potentials during charge and discharge were calculated. The Cu_1_Ni_1_–PCN-catalyzed cell exhibits the lowest overpotential and the highest *D*_Li_^+^, demonstrating its capability to enhance reaction kinetics and Li^+^ diffusion, thereby accelerating sulfur conversion [25]. Additionally, the impedance changes were obtained by fitting *in situ* electrochemical impedance spectroscopy (EIS; Fig. S13). Throughout the charge–discharge process, the Cu_1_Ni_1_–PCN-based cathode maintained consistently low resistance, demonstrating enhanced interfacial ion and electron transport. The resistance evolution at different voltages was further analysed using distribution of relaxation time (DRT) technology (Fig. 3d and Figs S14–S16) [26,27]. The C4 and D4 (1–10 s) peaks correspond to the ionic transport resistance across the cathode interface. Compared to the catalyst-free cathode, the intensity of the C4 peak in catalyst-containing cathodes increases during charging and decreases during discharging, which can be respectively attributed to the Li^+^ diffusion during the Li_2_S dissociation and the conversion of Li_2_S_2_ to Li_2_S. Notably, the Cu_1_Ni_1_–PCN-based cathode exhibited the lowest relaxation function γ (τ) values for both C4 and D4 peaks throughout the process (Fig. 3e), confirming its role in facilitating Li^+^/e^−^ transport within the composite cathode and significantly enhancing the overall reaction kinetics.

Temperature-dependent CV measurements were further conducted to determine the *E_a_* values for the oxidation and reduction processes with different catalysts (Fig. 3f) [28]. Notably, all three systems exhibit relatively low *E_a_* before cycling, highlighting the excellent activity of atomically dispersed catalysts in solid-state sulfur conversion. Among them, Cu_1_Ni_1_–PCN exhibits the lowest *E_a_* across the entire process, demonstrating its superior catalytic activity. Furthermore, DFT calculations provide deeper insights into the high catalytic activity of the BAC. Compared to Cu_1_–PCN and Ni_1_–PCN, the *d*-orbital center of Cu and Ni in Cu_1_Ni_1_–PCN shifts closer to the Fermi level, which enhances orbital hybridization and charge transfer with sulfur species, thereby accelerating solid-state sulfur conversion (Fig. 3g) [29]. Consistently, the adsorption energies of representative sulfur intermediates (Li_2_S_2_ and Li_2_S) reveal stronger binding interactions on Cu_1_Ni_1_–PCN relative to its single-metal counterparts (Fig. 3h, Fig. S17 and Table S3), confirming that the optimized *d*-band electronic structure directly translates into enhanced sulfur affinity and catalytic activity. In addition, the electronic redistribution between the two metal sites enables adaptive electron transfer, dynamically modulating their electronic structures during the redox process. Such synergistic electron buffering not only prevents the site-specific over-reduction or over-oxidation that often leads to catalyst deactivation but also ensures a persistent supply of catalytically active states throughout cycling. The catalytic advantage of this mechanism is further validated by the calculated Gibbs free energy profiles of the stepwise sulfur reduction reaction (Fig. 3i) [30]. In the rate-determining step from Li_2_S_2_ to Li_2_S [6], Cu_1_Ni_1_–PCN exhibits a significantly lower reaction free energy compared to Cu_1_–PCN and Ni_1_–PCN, rendering the transformation thermodynamically more favourable and kinetically more accessible. These results collectively demonstrate that the dual-metal coordination environment simultaneously optimizes reaction energetics and charge-transfer kinetics, thereby maximizing catalytic performance in solid-state sulfur conversion.

**Figure 4.**
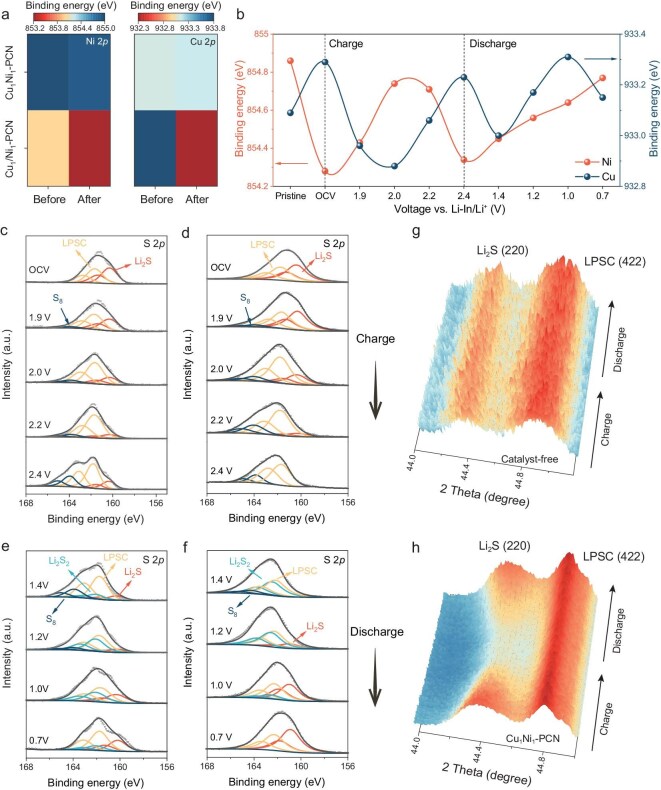
Stability of different catalysts in sulfur electrocatalysis and sulfur conversion kinetics. (a) Cu 2*p* and Ni 2*p* XPS spectra of Cu_1_–PCN, Ni_1_–PCN and Cu_1_Ni_1_–PCN before and after reaction. The data are presented as the values of the peak positions. (b) The changes in the peak positions of Cu and Ni in the XPS spectra of Cu_1_Ni_1_–PCN during the charge and discharge processes. *Ex situ* XPS spectra of the 2*p* level of S for batteries without catalyst (c and e) and those catalyzed by Cu_1_Ni_1_–PCN (d and f) after charging and discharging to a specific state. *In situ* XRD patterns of Li_2_S (220) evolution in battery without catalyst (g) and with Cu_1_Ni_1_–PCN (h) during the charge–discharge process.

Next, the stability of the catalysts was systematically evaluated. CV at a scan rate of 0.1 mV s^−1^ reveals distinct differences in durability among the catalysts. The cathodes containing Cu_1_–PCN and Ni_1_–PCN exhibited a significant decrease in peak current after 100 cycles (Fig. S18a and S18b). In contrast, the Cu_1_Ni_1_–PCN cathode demonstrated minimal variation, suggesting superior stability (Fig. S18c). To gain quantitative insight into this difference, temperature-dependent CV measurements were employed to determine the *E_a_* values of the oxidation and reduction processes after 100 cycles (Fig. 3f and Figs S19 and S20). Taking the reduction process as an example, Cu_1_Ni_1_–PCN maintained an almost constant *E_a_* before and after cycling (from 0.107 to 0.128 eV). However, significant increases in *E_a_* were observed for Cu_1_–PCN (from 0.122 to 0.246 eV) and Ni_1_–PCN (from 0.166 to 0.284 eV) after cycling. These results indicate the progressive degradation of SACs in the absence of an electron-buffering mechanism. The underlying origin of this stability disparity was further probed by *ex situ* XPS (Fig. 4a and Fig. S21). After 100 cycles, Cu and Ni in Cu_1_Ni_1_–PCN maintain stable valence states, ensuring sustained catalytic activity. In contrast, the Cu 2*p* and Ni 2*p* spectra of Cu_1_–PCN and Ni_1_–PCN gradually shift toward lower binding energies, reflecting electron over-accumulation and incomplete recovery of the original coordination structure. This irreversible change arises from the lack of a continuous electron supply once single-metal sites donate electrons to sulfur species, ultimately impairing their redox reversibility and long-term functionality. To directly monitor the dynamic redox evolution of the BAC during operation, *ex situ* XPS was carried out at different voltages within the first discharge–charge cycle (Fig. 4b and Fig. S22). At the initial stage of charging, strong interactions between sulfur species in Li_2_S and Ni sites facilitate electron transfer from S to Ni. During this process, Cu atoms functioned as electron buffers, accepting excess electrons from Ni to prevent its over-oxidation. As the charging progresses to 2.4 V, the Ni 2*p* peak shifts toward lower binding energy, indicating an electron-rich state, whereas the Cu 2*p* peak shifts toward higher binding energy, suggesting an electron-deficient state. Upon discharge, Ni adaptively transfers electrons back to Cu, followed by a concerted electron transfer from both metal sites to sulfur species, thereby accelerating the sulfur reduction process. At 0.7 V during full discharge, the desorption of reaction products restores the Cu and Ni binding energies close to their initial states. The calculated Bader charge evolution (Fig. S23) closely matches the valence-state shifts observed in XPS, confirming the consistency between the theoretical prediction and experimental evidence. This dynamic electron transfer between Cu and Ni effectively compensates for the substantial energy consumption associated with the structural reconfiguration of Cu–Ni sites during the reaction, thereby maintaining the catalyst’s high activity and long-term stability.

The *ex situ* S 2*p* XPS spectra provide further insights into the solid-phase sulfur transformation kinetics (Fig. 4c–f). Upon charging to 1.9 V, the binding energy of S_8_ at 164.1 eV begins to emerge. As the voltage increases to 2.4 V, the S_8_ signal is significantly intensified in the presence of Cu_1_Ni_1_–PCN, while the Li_2_S signal (160.1 eV) completely disappears. In contrast, in the catalyst-free battery, a substantial amount of Li_2_S remains, with only a small fraction converted to S_8_ [31,32]. Consistent with previous reports [6], the sulfur reduction pathway in the discharge follows the sequence S_8_ → Li_2_S_2_ → Li_2_S. At 1.2 V during discharge, S_8_ in the Cu_1_Ni_1_–PCN system is fully converted to Li_2_S_2_ (161.8 eV), with a minor fraction further reduced to Li_2_S. Upon complete discharge at 0.7 V, the catalyst-free system still retains a significant amount of Li_2_S_2_ intermediates, whereas Cu_1_Ni_1_–PCN enables the full conversion to Li_2_S. These observations confirm that the BAC effectively accelerates sulfur conversion kinetics, thereby enhancing the electrochemical performance. The *in situ* XRD analysis presented in Fig. 4g and h further demonstrates that the Cu_1_Ni_1_–PCN catalyst significantly enhances the sulfur conversion kinetics in the battery [33]. During the charging process, the Li_2_S (220) peak disappears at an earlier stage and completes its transformation at a lower potential in the presence of Cu_1_Ni_1_–PCN, indicating a more efficient oxidation process. Additionally, during discharge, the Li_2_S (220) peak emerges earlier, and the final amount of Li_2_S formed closely matches the initial content in the cathode, highlighting the catalyst’s ability to promote the reversible conversion of solid-phase sulfur. In contrast, the catalyst-free system exhibits only weak variations in the Li_2_S signal, suggesting sluggish sulfur conversion kinetics.

### Electrochemical performance of BAC

**Figure 5.**
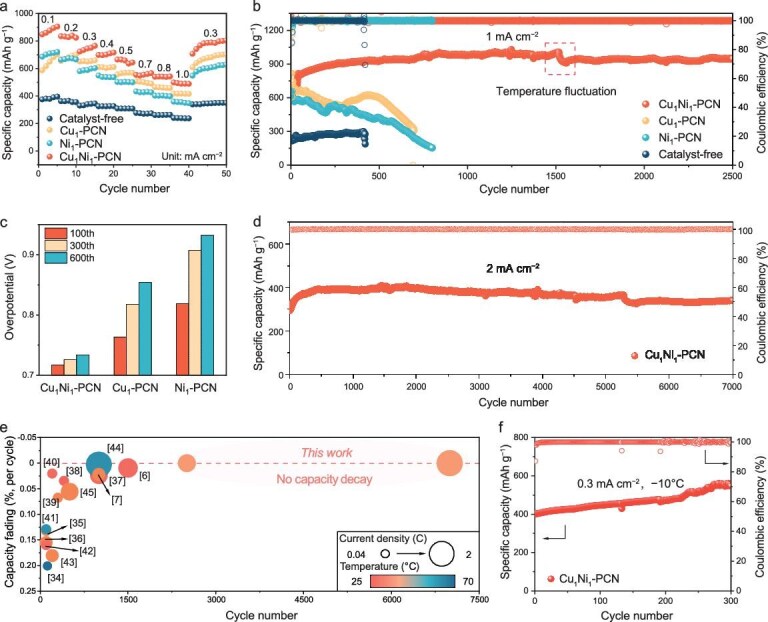
Electrochemical performance of ASSLSBs in the presence of Cu_1_Ni_1_–PCN catalyst. (a) Rate performance at different specific currents (from 0.1 to 1 mA cm^−2^). (b) Cycling stability of ASSLSBs with different cathodes at 1 mA cm^−2^. (c) The overpotentials of cells cycled at 1 mA cm^−2^ with various catalysts over different cycle numbers. (d) The cycling performance of the battery with Cu_1_Ni_1_-PCN at 2 mA cm^−2^. (e) Performance comparison between this work and previously reported ASSLSBs. (f) Cycle performance of Cu_1_Ni_1_–PCN-catalyzed ASSLSB at 0.3 mA cm^−2^ (−10°C).

We further validated the electrochemical performance of ASSLSBs assembled with different catalysts. As shown in Fig. 5a and Fig. S24, the battery with a Cu_1_Ni_1_–PCN cathode shows the highest discharge capacity across the current densities from 0.1 to 1.0 mA cm^−2^, confirming its excellent rate capability. Importantly, when the current density is restored to 0.3 mA cm^−2^, the cathode maintained a high specific capacity of ∼801 mAh g^−1^, demonstrating its exceptional reversibility and structural integrity of the BAC during repeated cycling. Figure 5b illustrates the long-term cycling performance of ASSLSBs at a current density of 1.0 mA cm^−2^ with a sulfur loading of approximately 1 mg cm^−2^. The catalyst-free battery rapidly fails, showing both the lowest discharge capacity and the shortest cycle life. Cathodes with Cu_1_–PCN and Ni_1_–PCN exhibit moderate improvements but still undergo pronounced capacity fading after ∼500 cycles. This degradation is consistent with the XPS observations of irreversible valence shifts, confirming that single-metal sites gradually deactivate due to the absence of a persistent electron-buffering pathway. In contrast, the Cu_1_Ni_1_–PCN cathode maintains an ultralow capacity decay, while retaining a high reversible capacity of 946.4 mAh g^−1^ over 2500 cycles. This remarkable cycling stability directly reflects the adaptive electron redistribution between Cu and Ni sites, which prevents local over-reduction or over-oxidation and preserves the active coordination structure. In addition, the battery with Cu_1_Ni_1_–PCN consistently maintained a lower overpotential throughout prolonged cycling, whereas the overpotentials of batteries with Cu_1_–PCN and Ni_1_–PCN increased significantly with cycle number. This further confirms the superior catalytic activity and stability of the BAC (Fig. 5c and Fig. S25).

Furthermore, the battery with Cu_1_Ni_1_–PCN exhibited excellent cycling stability at a high current density (2 mA cm^−2^), with an initial capacity increase followed by long-term stabilization. This is attributed to the inevitable electrode polarization at high current densities, which initially suppresses capacity. However, as cycling progresses, polarization gradually diminishes, enhancing the utilization of active materials and ultimately improving capacity retention. Remarkably, after 7000 cycles, negligible capacity decay was observed (Fig. 5d), highlighting its exceptional durability. These findings confirm that BAC effectively enhances the conversion kinetics of solid-phase sulfur species, enabling ASSLSBs to achieve high capacity and exceptional stability under high-rate and long-term cycling conditions. In Fig. 5e and Table S4, we further compared the electrochemical performance of our ASSLSBs with previously reported results[6,7,34–45], showing that our work significantly outperforms recent studies, which typically exhibit higher capacity decay rates, even at lower current densities. Finally, the robustness of the catalyst under harsh conditions was examined. Figure S26 reveals that at a low temperature of −10°C, the charge–discharge profiles of the battery exhibit a high overpotential, primarily due to the sluggish charge transfer kinetics within the electrode. Nevertheless, the battery maintains excellent reversibility, delivering a stable reversible discharge capacity of 547 mAh g^−1^ over 300 cycles (Fig. 5f), demonstrating its ability to sustain efficient sulfur conversion, even at low temperatures. Preliminary tests on an ASSLSB incorporating Cu_1_Ni_1_–PCN at an Li_2_S loading of 4.4 mg cm^−2^ demonstrate stable cycling at 0.1 mA cm^−2^ (Fig. S27), indicating that the electron-buffering strategy retains effectiveness under high-sulfur-loading conditions. These results confirm that the incorporation of BAC not only accelerates solid-phase sulfur conversion kinetics but also provides a robust electron-buffering mechanism that suppresses catalyst deactivation. This synergistic effect enables ASSLSBs to simultaneously achieve high capacity, ultra-long lifespan and reliable operation under demanding conditions, thereby representing a significant step forward in the practical development of high-performance ASSLSBs.

## CONCLUSION

In summary, our work proposes an atomic-scale electron buffering strategy that leverages adaptive electron transfer between dual-metal sites with comparable electronegativity. This mechanism shifts the *d*-band centers of the metal sites closer to the Fermi level, thereby strengthening their hybridization with the *p*-orbitals of sulfur species and reinforcing interfacial electronic interactions. Consequently, sufficient catalytic centers are generated at the confined solid–solid interface to accelerate sulfur conversion, while its dynamic valence-state modulation ensures the long-term activity and stability of the active sites. The battery with Cu_1_Ni_1_–PCN consistently maintains a low *E_a_* throughout long-term cycling, whereas the *E_a_* of the batteries with Cu_1_–PCN and Ni_1_–PCN increases significantly, indicating the superior catalytic stability of the BAC system. As a result, the ASSLSB with Cu_1_Ni_1_–PCN demonstrates remarkable cycling stability, maintaining stable operation over 7000 cycles, even at a high current density of 2 mA cm^−2^, while exhibiting robust cycling stability at −10°C. Overall, this study provides a promising strategy for designing highly active and durable metal catalysts to develop next-generation high-performance and long-lifespan ASSLSBs.

## MATERIALS AND METHODS

### Synthesis of Cu_1_Ni_1_–PCN

NiCl_2_·6H_2_O, CuCl_2_·2H_2_O and PCN were dispersed in ethanol solution, followed by ultrasonication for 30 min and subsequent rotary evaporation to remove the solvent [46]. The resulting solid was then dried at 80°C and heated to 300°C at a heating rate of 5°C min^−1^ under an argon atmosphere, maintaining this temperature for 5 h. After thorough washing with a water/ethanol mixture, the dried powder underwent high-temperature annealing at 550°C for 5 h under argon protection, with a heating rate of 2°C min^−1^.

### Synthesis of the cathode material

A mixture of Ketjenblack (Jiangsu XFNANO Materials Tech Co., Ltd.), Li_2_S, catalyst and Li_6_PS_5_Cl (Guangdong Canrd New Energy Technology Co., Ltd.) with a weight ratio of 1:1.8:0.2:2 was placed into a 50 mL agate ball-milling jar containing 12 g of 5 mm zirconia balls in the argon atmosphere. The ball-milling process was conducted at 550 rpm for 10 h utilizing a high-speed ball-milling machine.

### Full cell assembly and electrochemical measurements

ASSLSBs were assembled in a custom-designed stainless-steel mold with a diameter of 10 mm (Beijing Zhongke Wanyuan Technology Co. Ltd.) by a cold pressing method inside an argon-filled glovebox. The pressurization equipment used was from the Shenzhen Kejing Star Technology company (YLJ-15T-LD). The lithium–indium alloy (Li–In) sheet was put on the negative side of the solid-state battery compression mold. Subsequently, the solid electrolyte Li_6_PS_5_Cl was introduced, and the pressure was elevated to 375 MPa before incorporating the sulfur composite electrode. After sealing, the assembly was further compressed to 100 MPa to form the full cell. The charge–discharge measurements were conducted using a Neware battery test system at room temperature. CV curves were obtained using an Ivium workstation at a scan rate of 0.1 mV s^−1^.

### GITT measurement

GITT tests for the full cell were conducted at a current density of 0.1 mA cm^−2^. By analyzing the relationship between the response potential and current relaxation time, along with the lithiation parameters of the active material, the ion diffusion coefficient within the electrode and the battery’s overpotential can be calculated [47–49].

### Computational details

DFT calculations were performed using the Vienna *Ab Initio* Simulation Package (VASP) with the generalized gradient approximation (GGA) method [50,51]. Electron–ion interactions were treated by the projector augmented-wave (PAW) method and the Perdew–Burke–Ernzerhof (PBE) functional was employed for describing the exchange and correlation energies [52]; the energy cutoff was set at 400 eV. The Brillouin zone was sampled by a Γ-centered k-point mesh of (1 × 1 × 1). In addition, van der Waals interactions (vdWs) between catalysts and sulfur species in the adsorption simulations were considered using the DFT + D_3_ method [53–55]. The energy convergence criterion between the two electronic steps was set to 10^−5^ eV, and the system structures before and after sulfur species adsorption were optimized until the Herman–Feynman force of each atom reached below 0.01 eV Å^–1^.

## Supplementary Material

nwag024_Supplemental_File
