# Supplementary material for: Dual-atom pairs with electron buffering enable ultrastable-cycling all-solid-state lithium–sulfur batteries
Source: Natl Sci Rev. 2026 Jan 15;13(4):nwag024. doi: 10.1093/nsr/nwag024 (PMC12900420; doi:10.1093/nsr/nwag024)
Supplement: nwag024_Supplemental_File [file nwag024_supplemental_file.pdf]

Supplementary materials for

Dual-atom pairs with electron buffering enable ultrastable cycling all-solid-state lithium-sulfur batteries

Jiwei Shi[1,#], Mingyang Jiang[1,#], Chuannan Geng[1], Zhonghao Hu[1], Yun Cao[1], Jiaqi Lan[1], Li Wang[2,3], Quan-Hong Yang[2,3] and Wei Lv[1*]

[1]Shenzhen All-Solid-State Lithium Battery Electrolyte Engineering Research Center, Key Laboratory of Electrocatalytic Materials and Green Hydrogen Technology of Guangdong Higher Education Institutes, Shenzhen Key Laboratory for Graphene-based Materials, Institute of Materials Research, Tsinghua Shenzhen International Graduate School, Tsinghua University, Shenzhen 518055, China;

[2]Nanoyang Group, Tianjin Key Laboratory of Advanced Carbon and Electrochemical Energy Storage, State Key Laboratory of Chemical Engineering and Low-Carbon Technology, School of Chemical Engineering and Technology, National Industry-Education Platform for Energy Storage, and Collaborative Innovation Center of Chemical Science and Engineering, Tianjin University, Tianjin 300072, China;

[3]Haihe Laboratory of Sustainable Chemical Transformations, Tianjin 300192, China

***Corresponding authors.** E-mails: qhyangcn@tju.edu.cn; lv.wei@sz.tsinghua.edu.cn

[#]Equally contributed to this work.

MATERIALS AND METHODS

Synthesis of PCN

Dicyandiamide (Macklin) was calcined at 550°C (heating rate, 2.3°C min^{-1}) for 4 h in a muffle furnace, after which it was thermally exfoliated at 500°C (heating rate, 5°C min^{-1}) for 5 hours to obtain the PCN nanosheets.

Synthesis of Cu_1-PCN and Ni_1-PCN

$NiCl_2 \cdot 6H_2O$ (200 mg) and PCN (500 mg) were dispersed in 40 mL of ethanol solution and ultrasonicated for 30 min, followed by rotary evaporation to remove the solvent. The resulting solid was then dried at 80°C and subsequently heated to 450°C at a heating of 5°C min^{-1} under an argon atmosphere, maintaining this temperature for 5 h. The obtained powder was thoroughly washed with a water-ethanol mixture and dried again at 80°C. Finally, the sample was heated to 550°C at a rate of 2°C min^{-1} under argon protection and held at this temperature for 5 h to obtain the final product.

Material characterization

Powder X-ray diffraction (XRD) patterns were carried out on a Bruker D8 Focus Powder X-ray diffractometer using Cu Kα radiation (λ = 0.154 nm) at room temperature. Scanning electron microscope (SEM) was performed on a Regulus 8100 (Hitachi Japan) instrument with an accelerating voltage of 5.0 kV. High-resolution transmission electron microscopy (HRTEM) observations were made on a JEM 2100F coupled with an energy dispersive spectrometer (EDS), aberration corrected transmission electron microscope (Thermo Fisher Scientific, Spectra 300) operating at 200 kV contributed to Spherical aberration-corrected high-angle annular dark-field scanning transmission electron microscope (HAADF-STEM) images. XPS measurements were made using a Thermo Fisher Scientific K-Alpha+ unit with Al Kα radiation, $h\nu$ = 1486.6 eV. X-ray absorption near edge structure (XANES) and extended X-ray absorption fine structure (EXAFS) measurements were carried out at the XAFCA beamline of the Singapore Synchrotron Light Source (SSLS). A Si (111) double-crystal monochromator was used

to filter the X-ray beam. Metal foils were used for the energy calibration, and all samples were measured under transmission mode at room temperature. The XAFS data were processed according to the standard procedures using the Athena module implemented in the IFEFFIT software packages. The EXAFS spectra were obtained by subtracting the post-edge background from the overall absorption and then normalizing with respect to the edge-jump step. Subsequently, the *χ(k)* data of were Fourier transformed to real (R) space using a hanning windows (dk = 1.0 $Å^{-1}$) to separate the EXAFS contributions from different coordination shells. To obtain the quantitative structural parameters around central atoms, least-squares curve parameter fitting was performed using the ARTEMIS module of IFEFFIT software packages. The in situ XRD tests used a Rigaku D/MAX 2500/PC X-Ray Diffractometer, with a 2 Theta range of 44°-46.5° and a scan rate of 0.5° min^{-1}. Cu-Kα (λ = 0.154 nm) radiation was used with an operating voltage of 40 kV. A specially designed airtight solid-state XRD cell was employed to handle the air-sensitive LPSC. The setup features a stainless-steel housing equipped with a 200 μm thick high-transmittance beryllium (Be) window, which directly contacts the composite cathode to enable efficient X-ray transmission while preserving gas tightness and sustaining a constant stack pressure of approximately 20 MPa. Operando measurements were conducted at a current rate of 0.05 mA cm^{-2} to monitor sulfur redox processes. To obtain reliable sulfur-conversion signals, the diffraction scan was confined to a narrow ~5° 2θ region centered on the characteristic reflection, combined with a fine scanning rate of ≤ 0.5° min^{-1}.

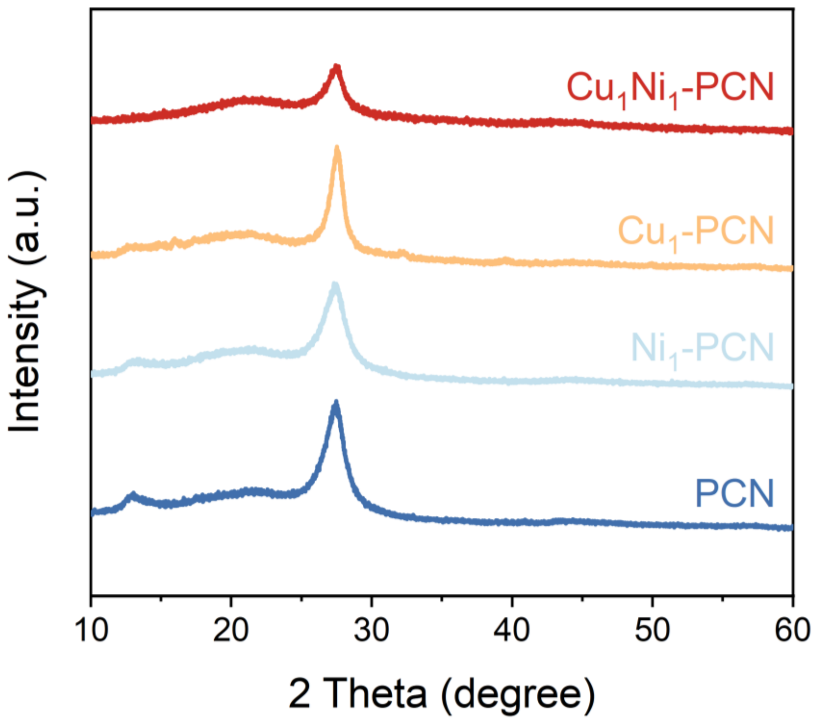

Figure S1 | XRD patterns of various catalysts.

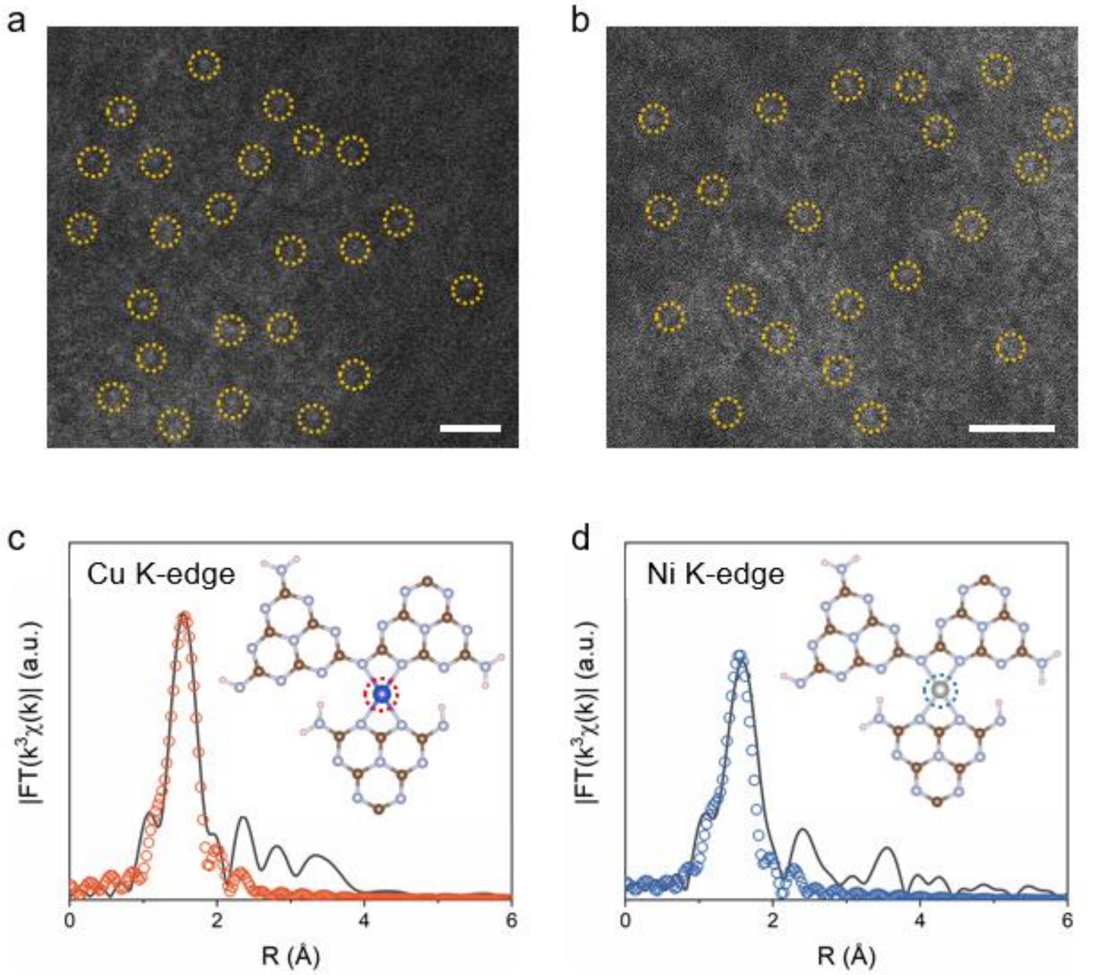

Figure S2 | a, b, Atomic-resolution ADF-STEM images of Cu_1-PCN and Ni_1-PCN (single atoms are circled). Scale bar, 1 nm. k^3-weighted Cu and Ni K-edge Fourier-transformed (FT) EXAFS spectra of Cu_1-PCN (**c**) and Ni_1-PCN (**d**).

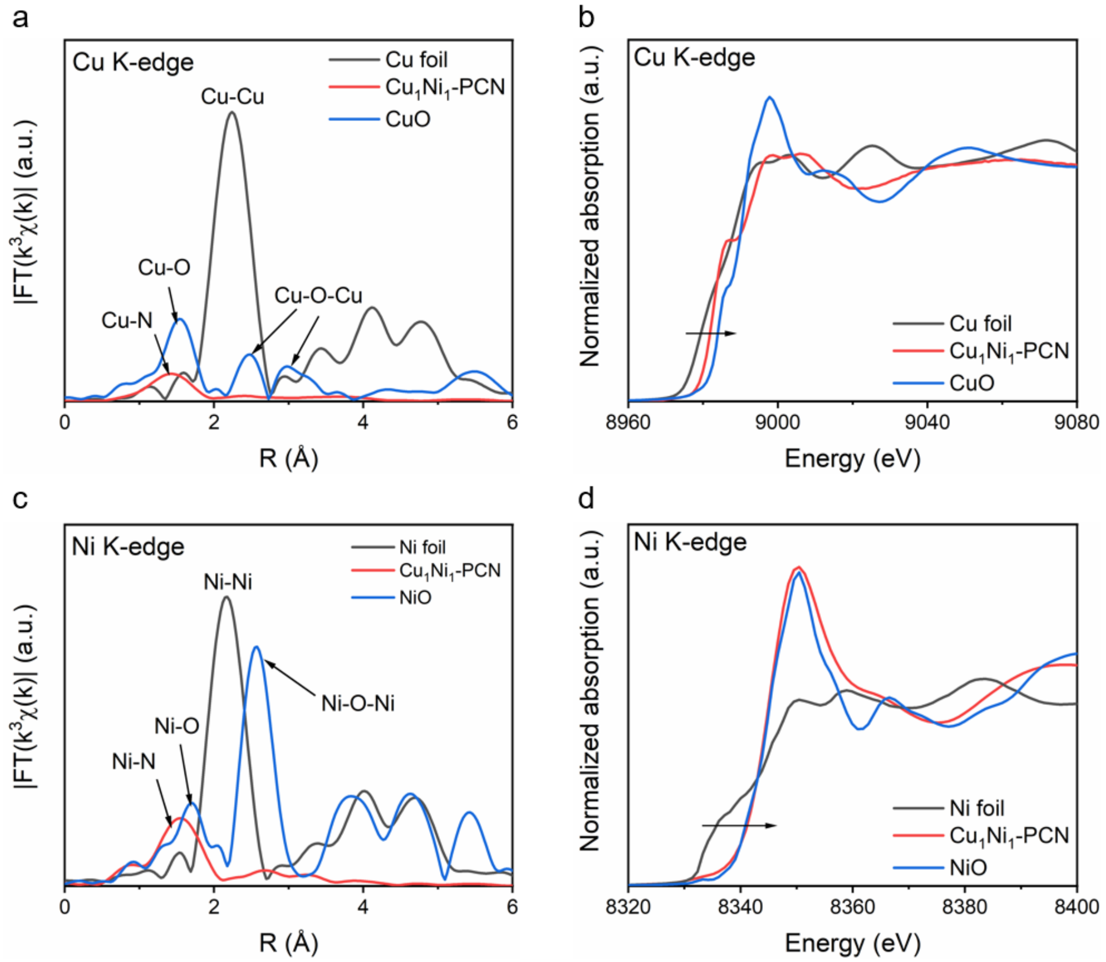

Figure S3 | k^3-weighted Cu K-edge FT-EXAFS spectra (**a**) and Cu K-edge X-ray absorption near edge structure (XANES) spectra for Cu_1Ni_1-PCN, Cu foil, and CuO (**b**). k^3-weighted Ni K-edge FT-EXAFS spectra (**c**) and Ni K-edge XANES spectra for Cu_1Ni_1-PCN, Ni foil, and NiO (**d**).

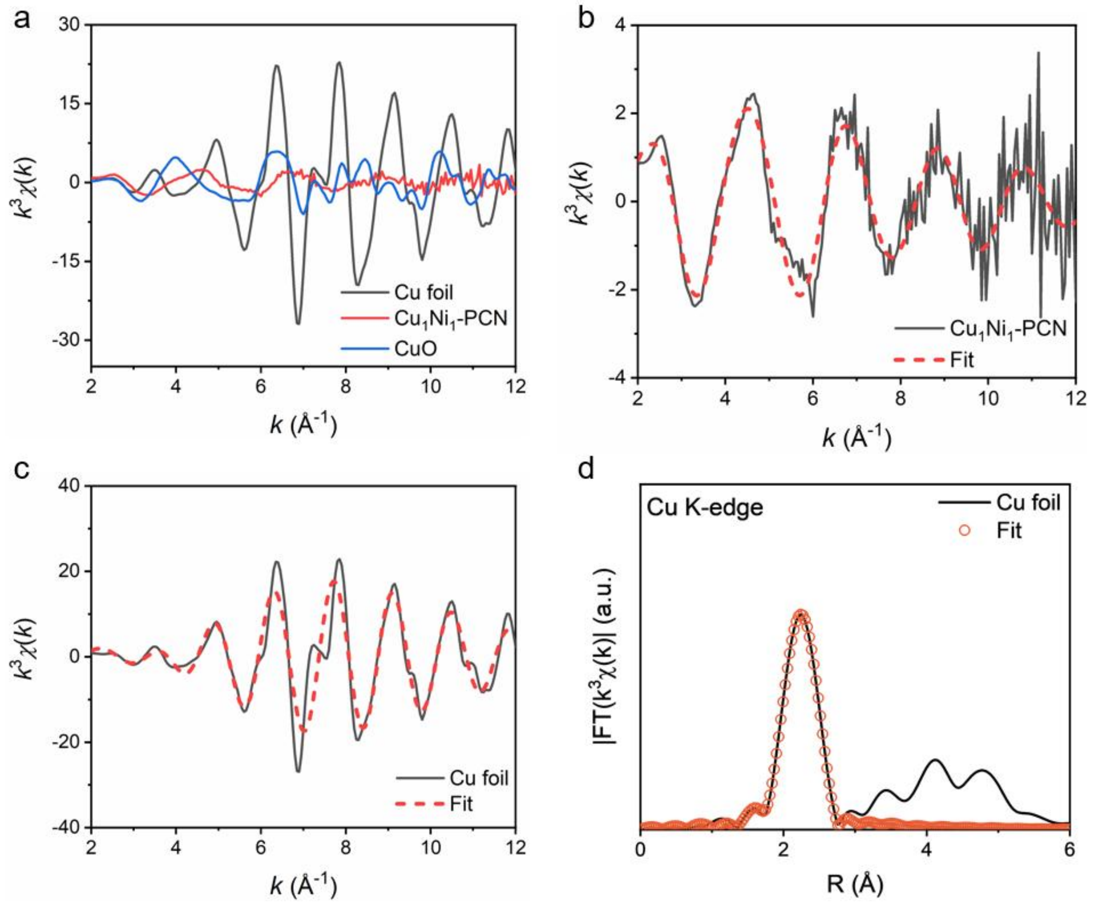

Figure S4 | a, FT-EXAFS in k space of Cu_1Ni_1-PCN, Cu foil, and CuO. **b-c,** experimental data and fitting of (**b**) Cu_1Ni_1-PCN, (**c**) Cu foil. **d,** Cu K-edge FT-EXAFS spectra of Cu foil.

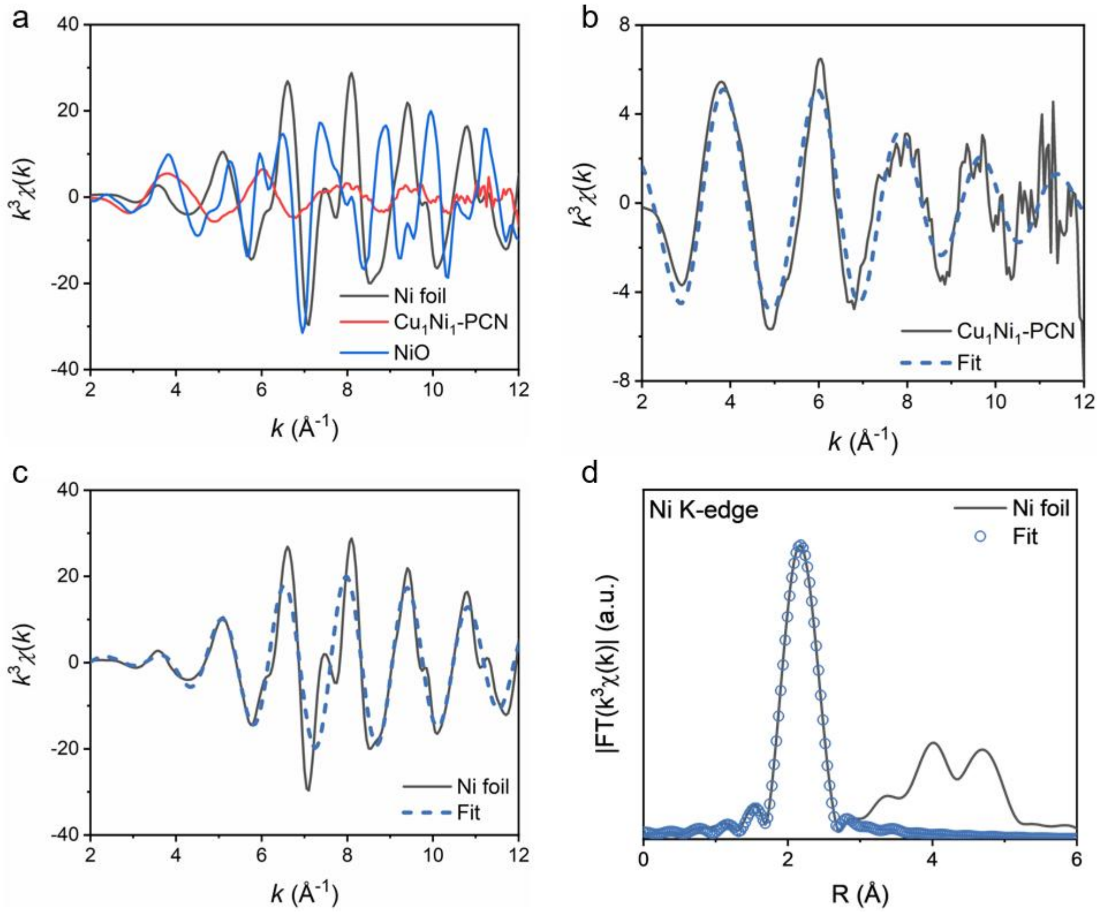

Figure S5 | a, FT-EXAFS in k space of Cu_1Ni_1-PCN, Ni foil, and NiO. **b-c,** experimental data and fitting of (**b**) Cu_1Ni_1-PCN, (**c**) Ni foil. **d,** Ni K-edge FT-EXAFS spectra of Ni foil.

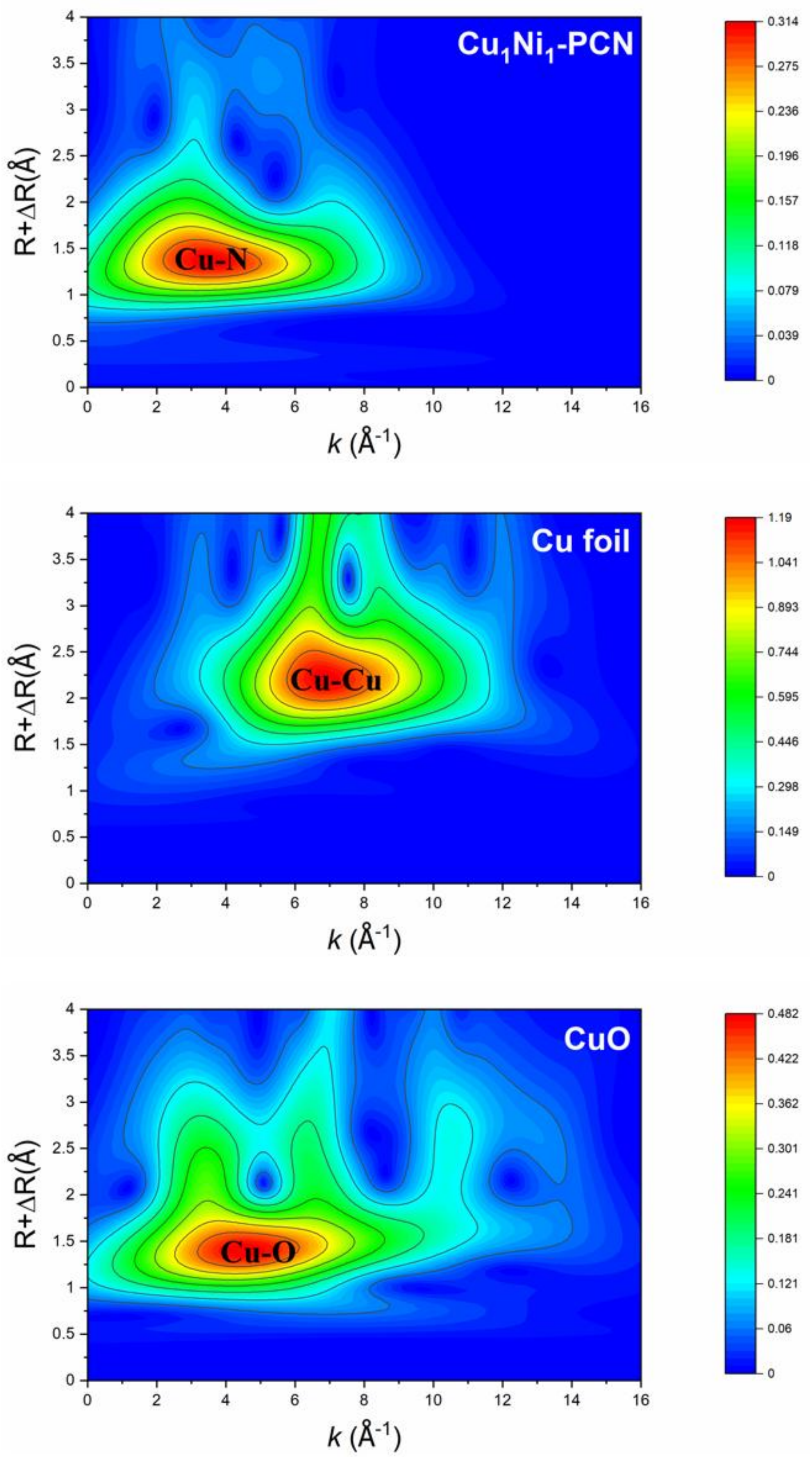

Figure S6 | Wavelet transform of Cu K-edge EXAFS spectra for Cu_1Ni_1-PCN, Cu foil, and CuO.

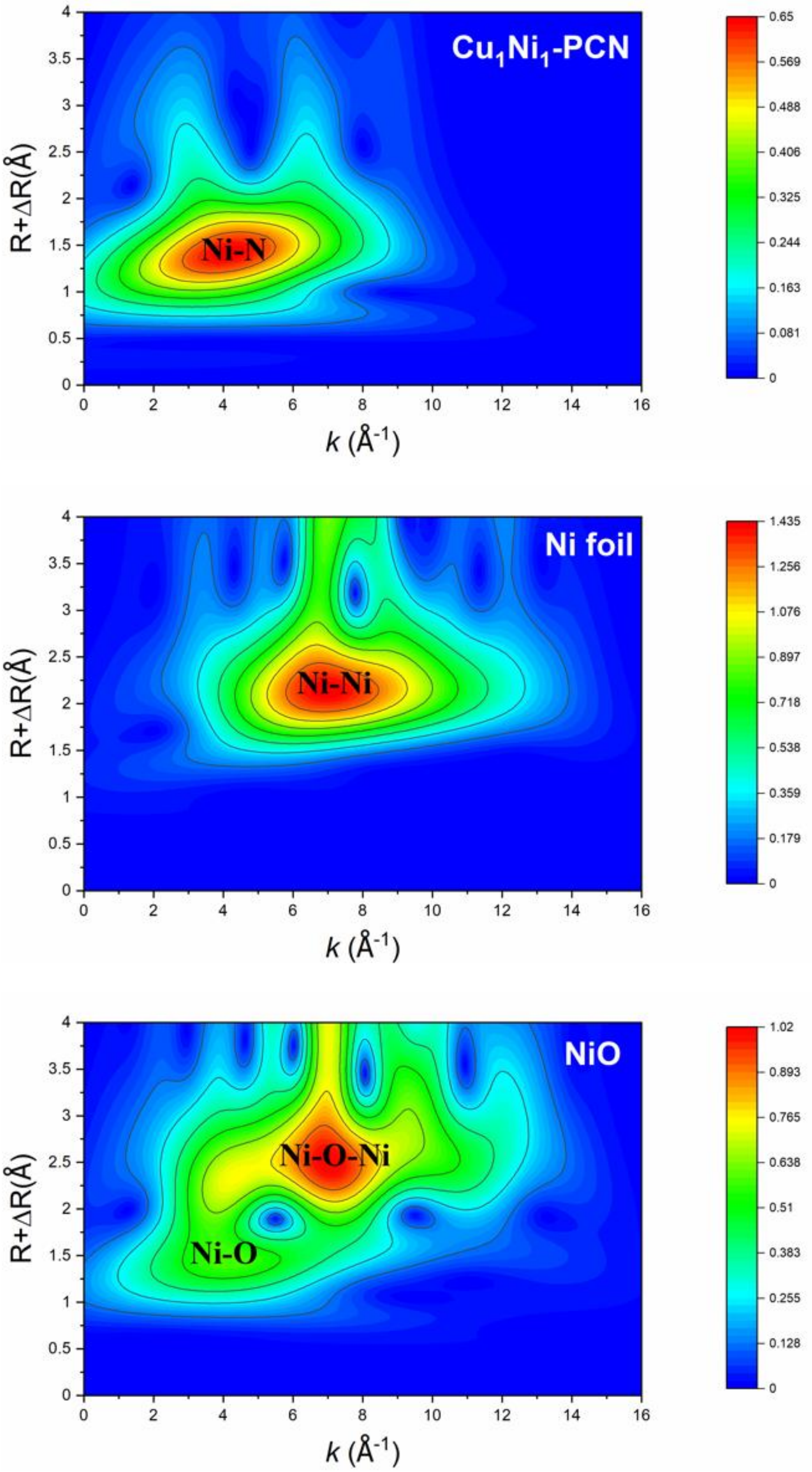

Figure S7 | Wavelet transform of Ni K-edge EXAFS spectra for Cu_1Ni_1-PCN, Ni foil, and NiO.

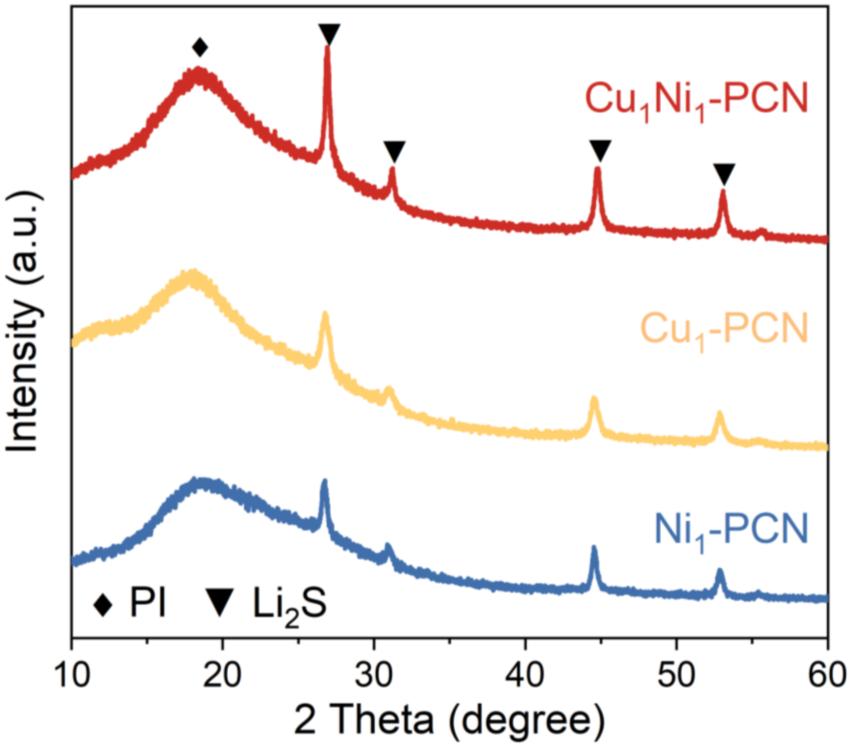

Figure S8 | XRD patterns of various cathodes.

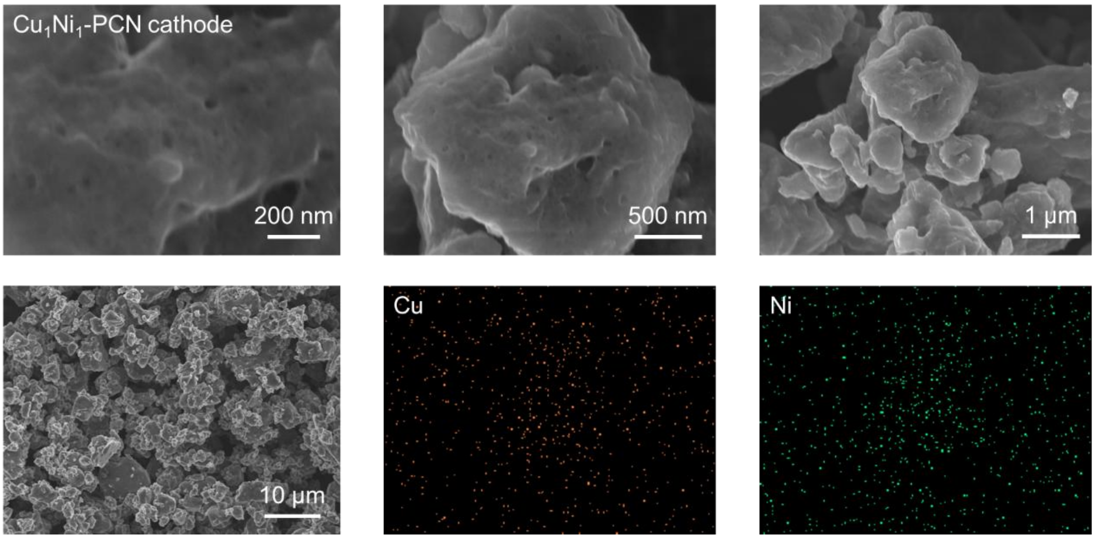

Figure S9 | SEM images and ESD maps of the cathode material with Cu_1Ni_1-PCN.

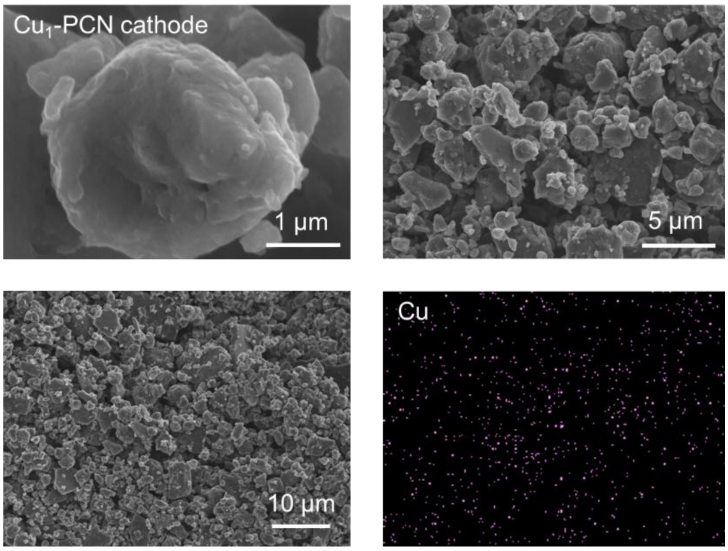

Figure S10 | SEM images and ESD maps of the cathode material with Cu_1-PCN.

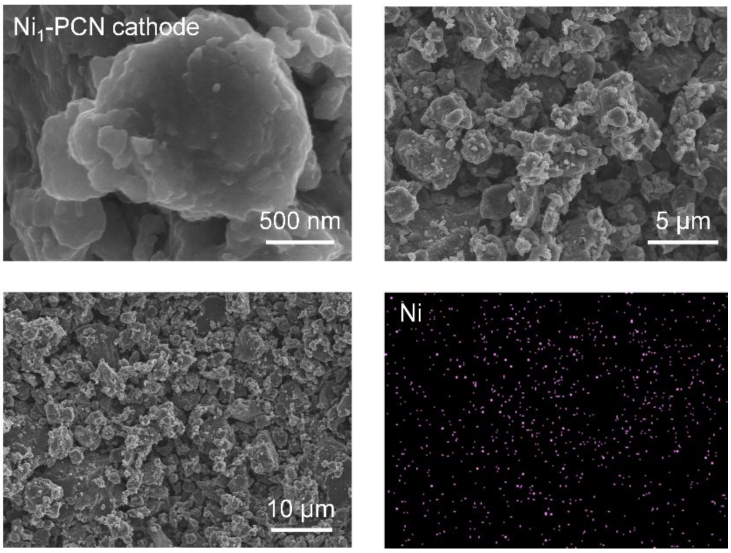

Figure S11 | SEM images and ESD maps of the cathode material with Ni_1-PCN.

The crystal structure of the materials obtained after ball milling was characterized using XRD (**Figure S8**), after ball milling, only the characteristic peak of Li_2S is detected in the cathode, and no obvious metal particle peak appeared, indicating that ball milling uniformly mixes the catalyst in the cathode material without changing the crystal structure of the catalyst. The microstructure of the cathode material with different catalysts was observed by scanning electron microscope (SEM). From the images, it can be observed that the overall morphology of the cathode material is unchanged after introducing different catalysts via high-energy ball milling (**Figures S9-11**). EDS maps indicate that catalysts are uniformly distributed in the cathode material without significant aggregation.

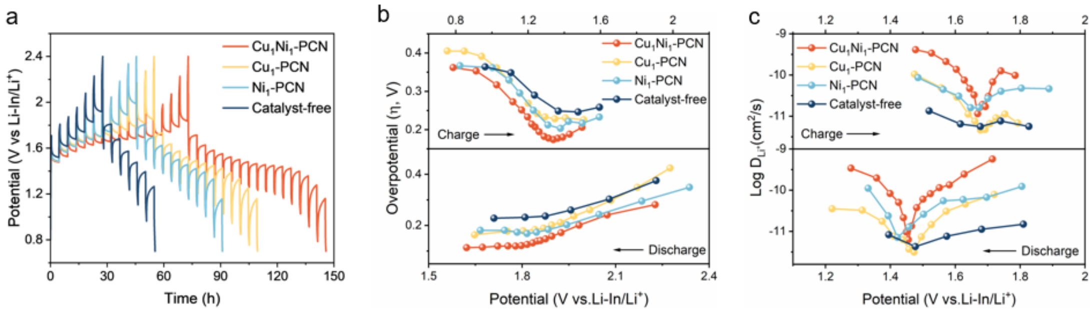

Figure S12 | a, GITT results of ASSLSBs with different cathodes. **b**, Overpotential of different cathodes under different potentials. **c**, Li^+ diffusion coefficient of composite cathodes containing different catalysts calculated from GITT curves.

During GITT testing, the constant current pulse signal is applied to the electrode under investigation. Each pulse cycle consists of two steps: a fixed period of "constant current charge/discharge" followed by a "rest" period. After a certain period of charge/discharge, the current is cut off, allowing the ions within the active material to diffuse until they reach equilibrium. By analyzing the relationship between the response potential and current relaxation time, along with the lithiation parameters of the active material, the ion diffusion coefficient within the electrode and the battery's overpotential can be calculated.

$$D = \frac{4d^2}{\pi\tau}\left(\frac{\Delta E_S}{\Delta E_t}\right)^2$$

Where, d (cm) represents the electrode thickness, τ (s) represents the relaxation time during the test, ΔE_S (V) is the voltage change during the resting process, and the voltage change during the constant current charge/discharge process is denoted as ΔE_t (V).

$$\eta = \Delta E_t + IR - \Delta E_S$$

Where, η (V) represents the overpotential, and IR (V) represents the voltage change because of the ohmic resistance of the electrode and interface when the current is applied.

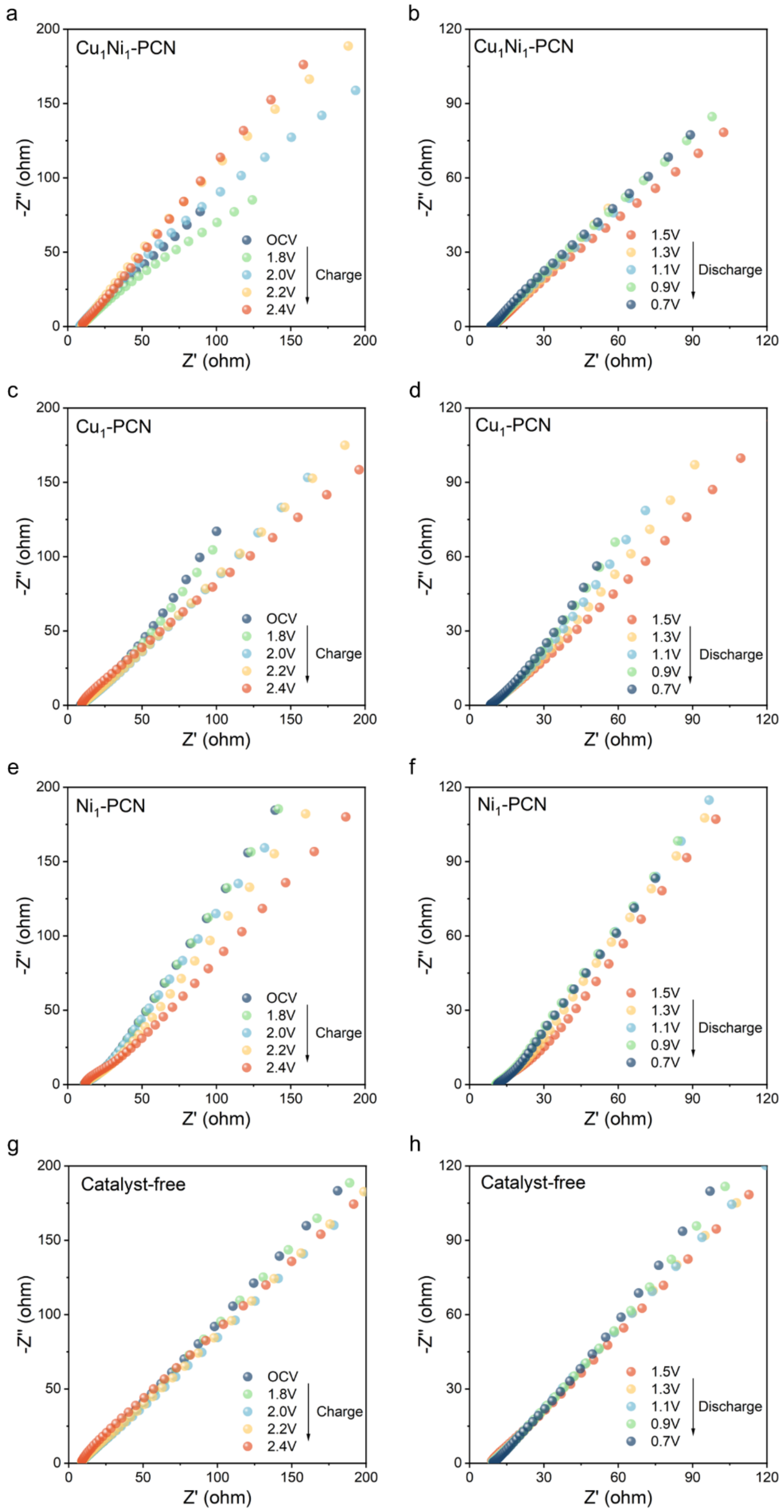

Figure S13 | In situ EIS plots for charge and discharge process with Cu_1Ni_1-PCN (**a-b**), Cu_1-PCN (**c-d**), Ni_1-PCN (**e-f**), and without catalyst (**g-h**).

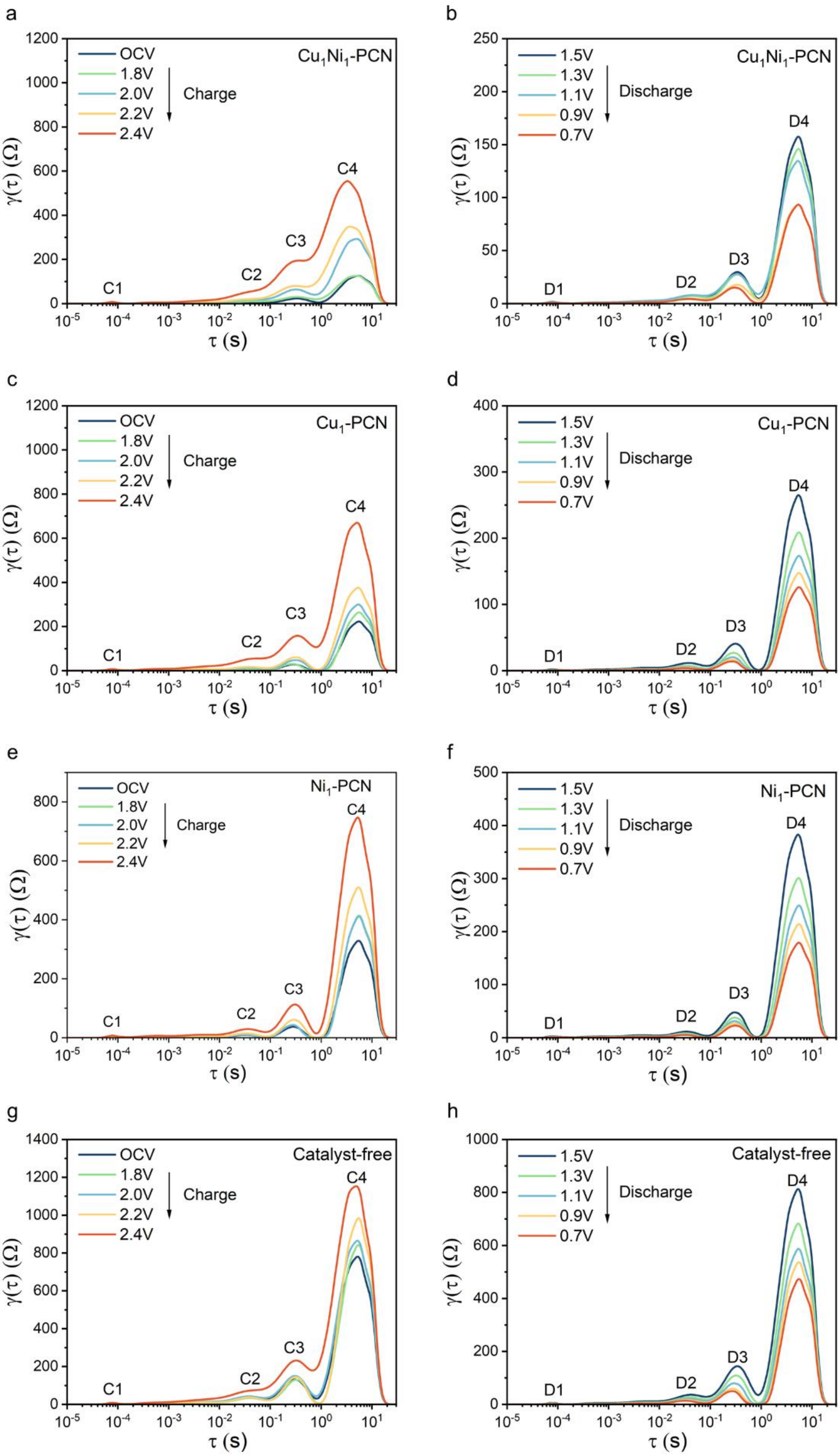

Figure S14 | DRT profiles of charge and discharge process for the cathode with Cu_1Ni_1-PCN (**a-b**), Cu_1-PCN (**c-d**), Ni_1-PCN (**e-f**), and without catalyst (**g-h**).

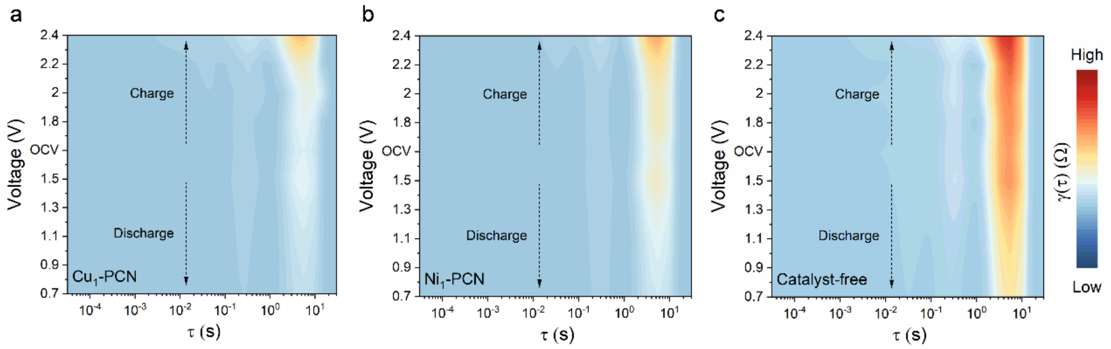

Figure S15 | The DRT curves plotted calculated from the in situ EIS results and the corresponding 2D intensity mapping of cathodes with Cu_1-PCN (**a**), Ni_1-PCN (**b**), and without catalyst (**c**).

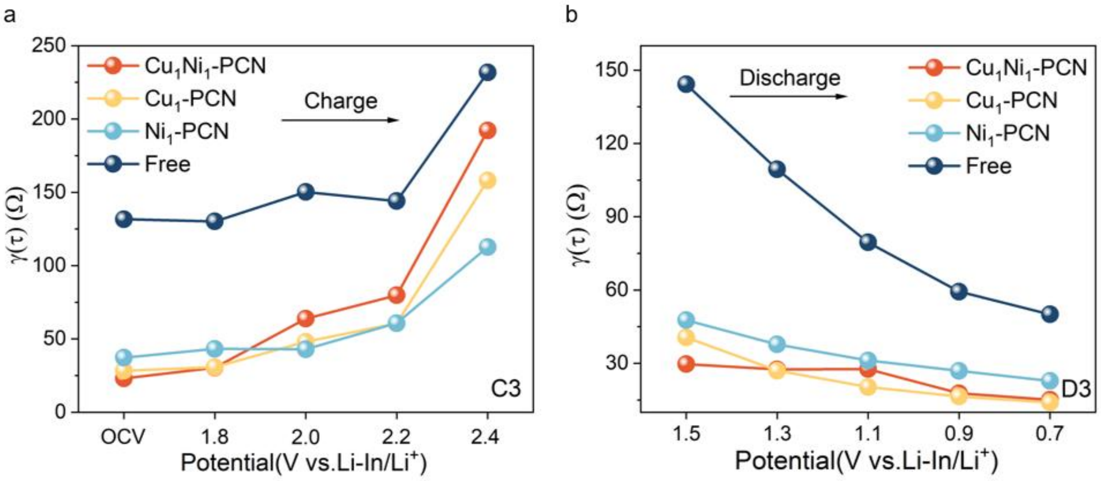

Figure S16 | Evolution of relaxation-based function γ(τ) of charge (**a**) and discharge (**b**) process for cathodes with Cu_1Ni_1-PCN, Cu_1-PCN, Ni_1-PCN and without catalyst.

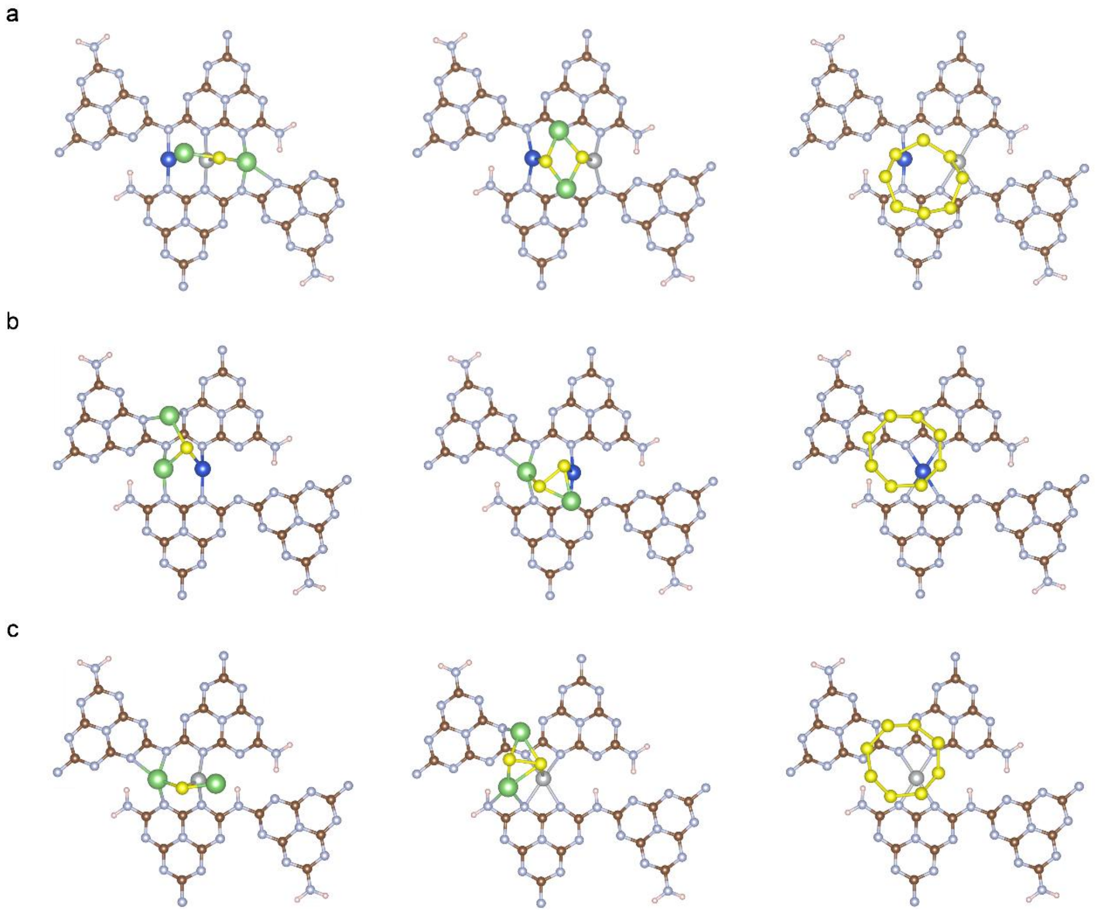

Figure S17 | Adsorption configurations of sulfur species on Cu_1Ni_1-PCN (**a**), Cu_1-PCN (**b**) and Ni_1-PCN (**c**) catalysts.

The adsorption energy (E_b) between the polysulfides and the catalyst was calculated by the following formula.

$$E_b = E_{total} - E_{catalyst} - E_{adsorbate}$$

where E_{total} is the of the configuration with sulfur species absorbed, $E_{catalyst}$ and $E_{adsorbate}$ are the energies of the pure catalyst and isolated sulfur species.

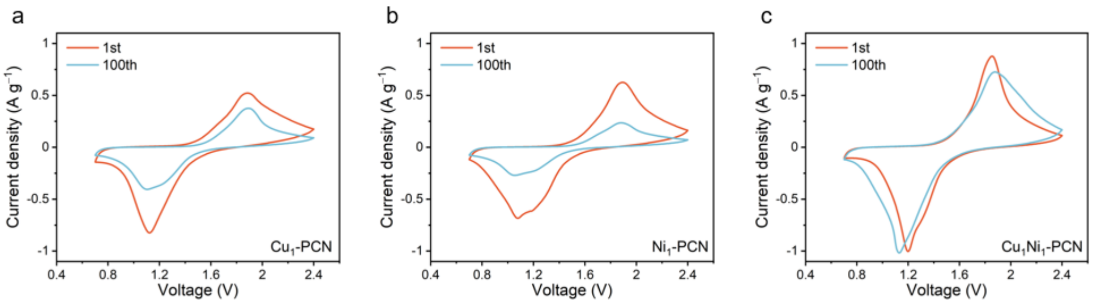

Figure S18 | Comparison of CV curves of batteries containing Cu_1-PCN (**a**), Ni_1-PCN (**b**) and Cu_1Ni_1-PCN (**c**) before and after 100 cycles.

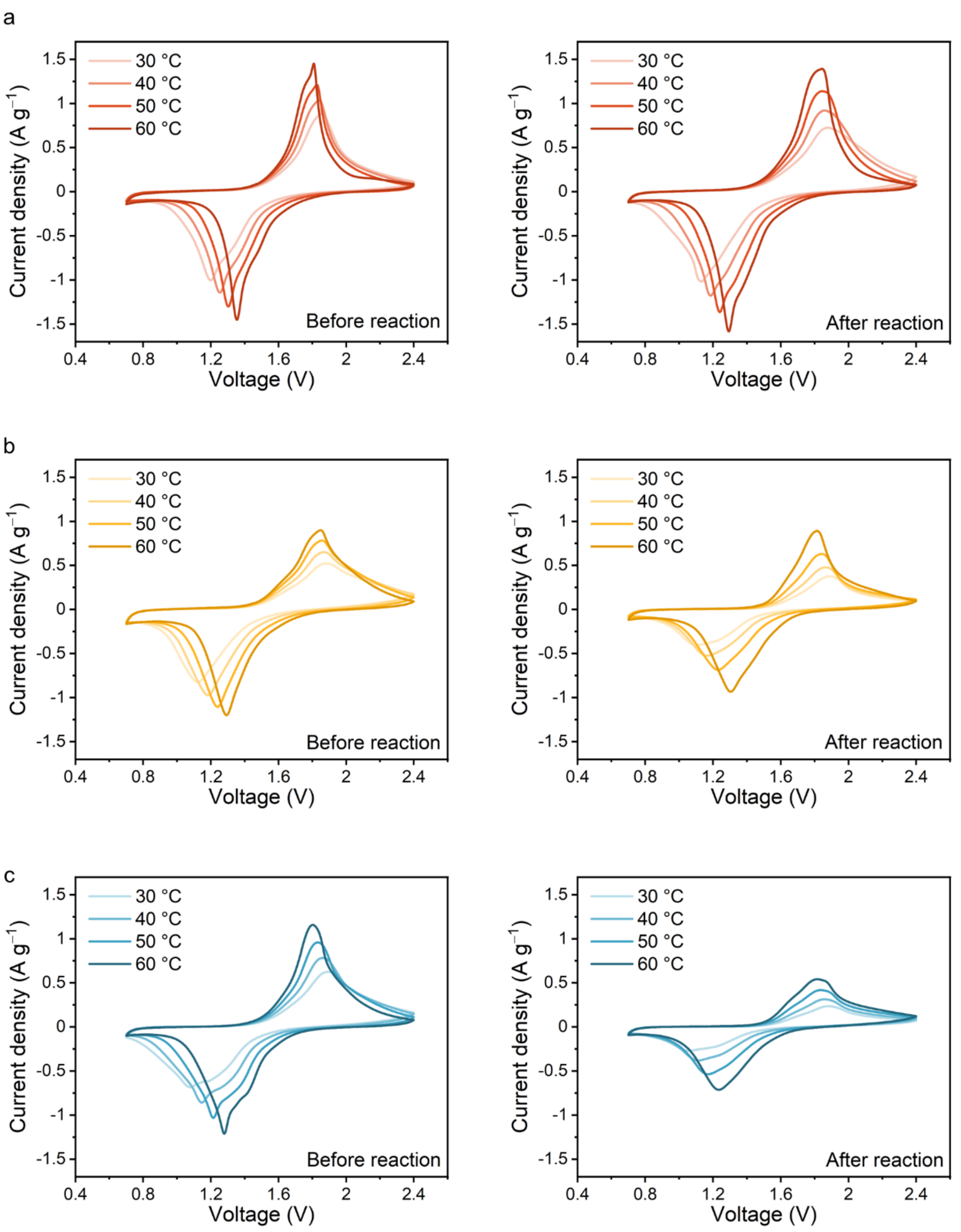

Figure S19 | CV profiles of different catalysts-catalyzed cells at different temperatures with a scan rate of 0.1 mV s^{-1}. Cu_1Ni_1-PCN (**a**), Cu_1-PCN (**b**) and Ni_1-PCN (**c**).

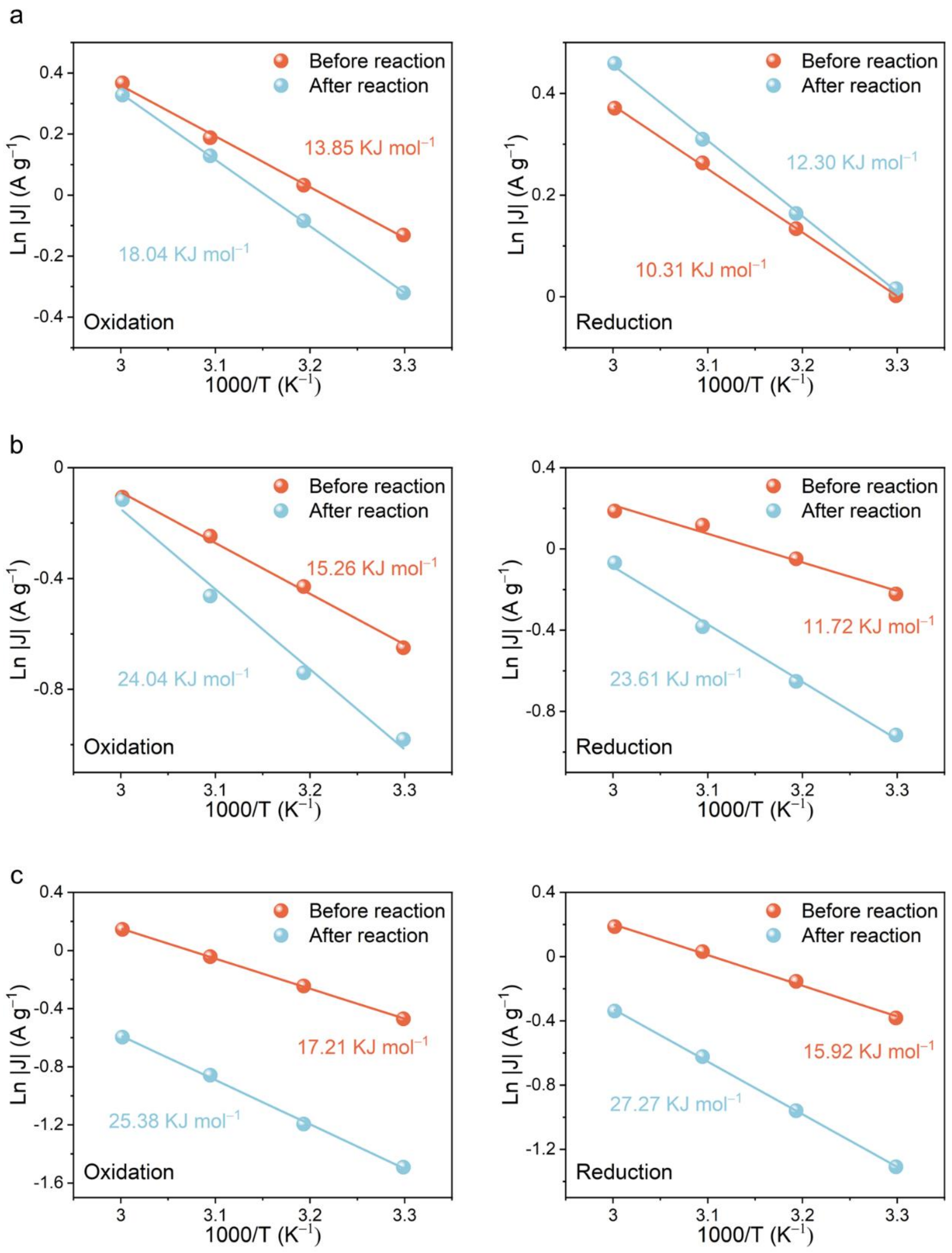

Figure S20 | Relationship between the peak current of redox process in CV curves and temperature for the cells with different catalysts: Cu_1Ni_1-PCN (**a**), Cu_1-PCN (**b**) and Ni_1-PCN (**c**).

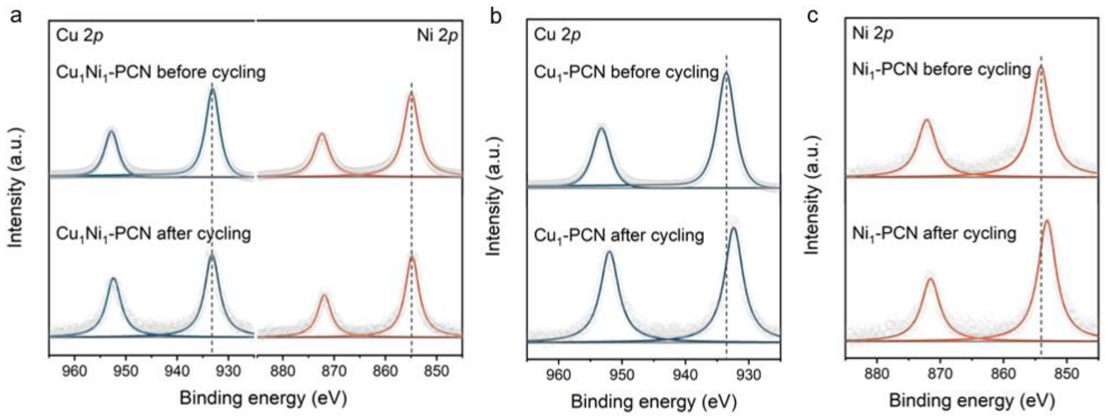

Figure S21 | Cu 2*p* and Ni 2*p* XPS spectra of Cu_1Ni_1-PCN (**a**), Cu_1-PCN (**b**) and Ni_1-PCN (**c**) before and after cycling.

Supplementary Note 1: The chemical reaction equation of the entire reaction process.

$$S_8 + 8\,Li^+ + 8\,e^- + 4\,* \rightarrow 4\,Li_2S_2^* \tag{1}$$

$$4\,Li_2S_2^* + 8\,Li^+ + 8\,e^- + 4\,* \rightarrow 8\,Li_2S^* \tag{2}$$

$$8\,Li_2S^* \rightarrow 8\,Li_2S + 8\,* \tag{3}$$

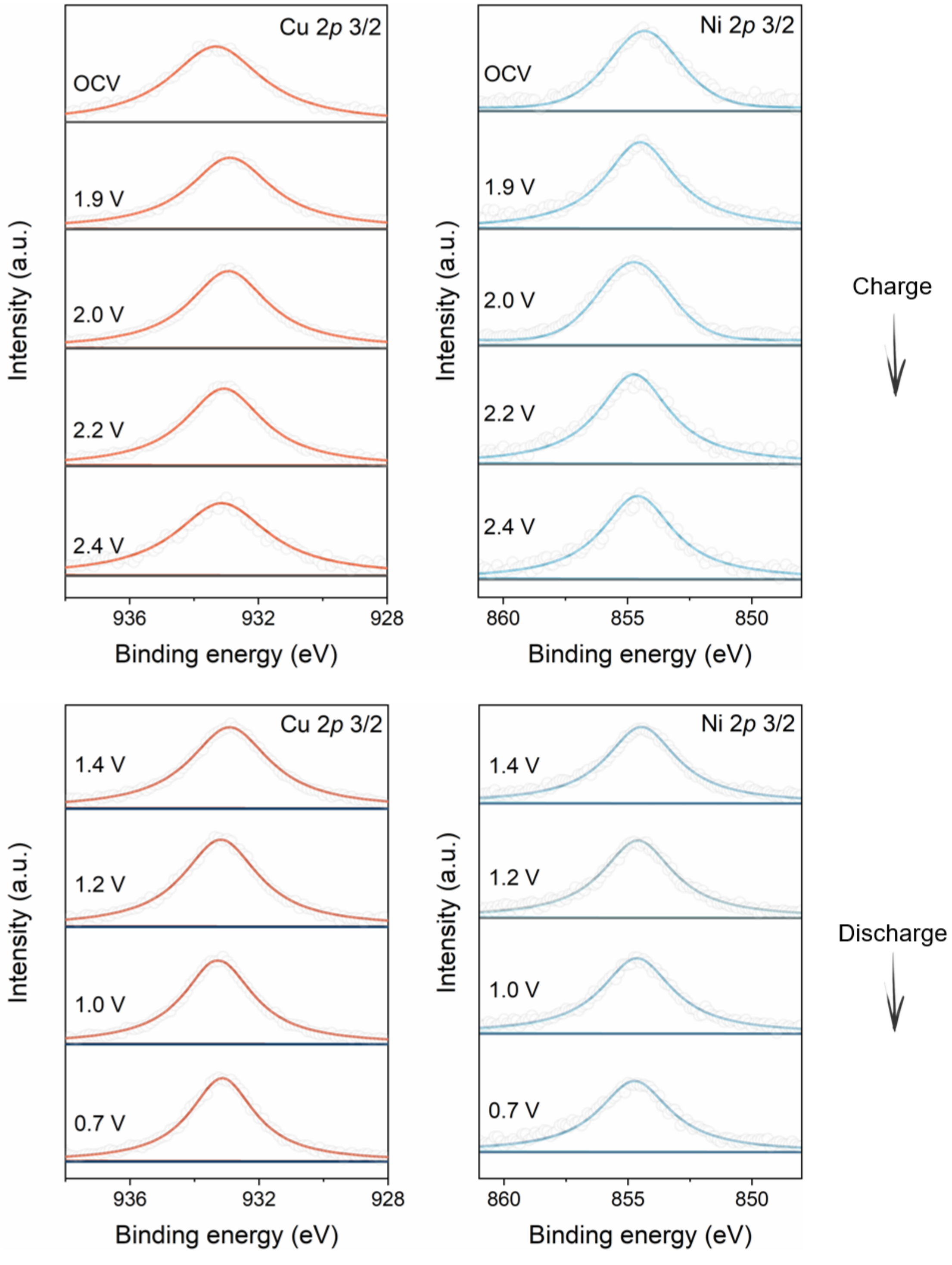

Figure S22 | Cu 2*p* and Ni 2*p* XPS spectra of Cu_1Ni_1-PCN during the first charge-discharge process.

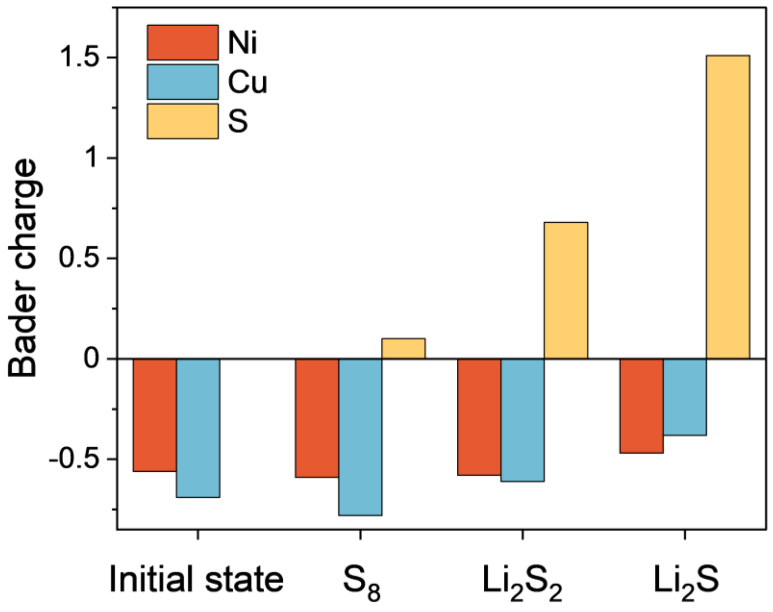

Figure S23 | Bader charge evolution of Cu_1Ni_1-PCN during its interactions with different sulfur species (S_8, Li_2S_2, and Li_2S).

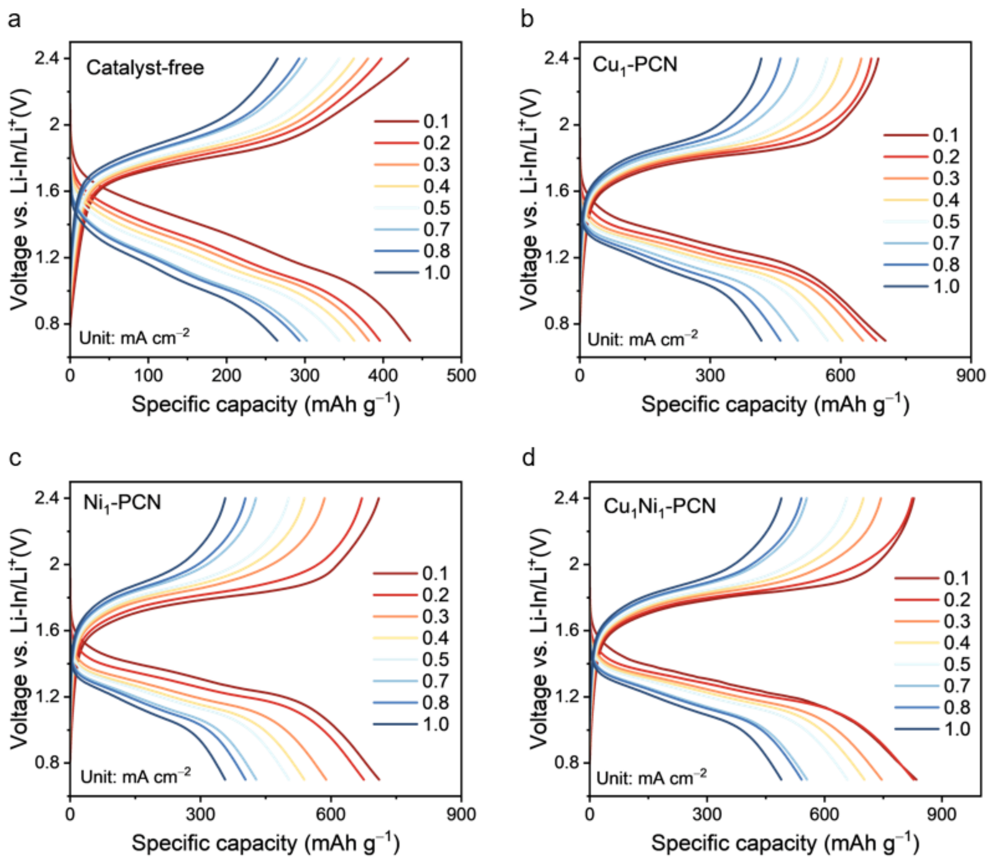

Figure S24 | Galvanostatic discharge-charge profiles at different specific currents in the presence of different catalysts.

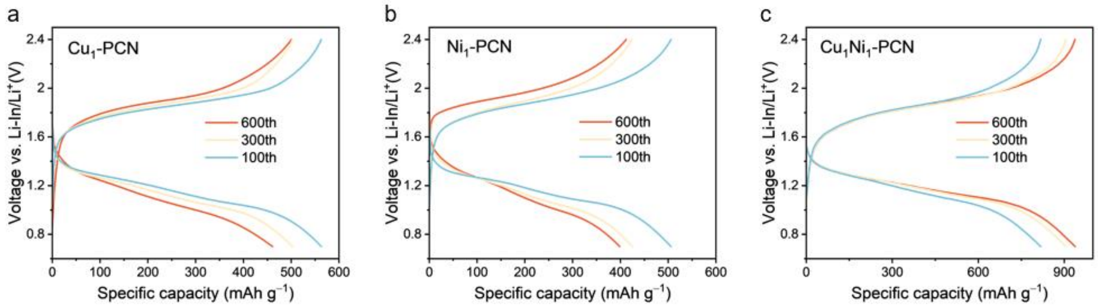

Figure S25 | Galvanostatic discharge-charge profiles of cells with various catalysts over different cycle numbers.

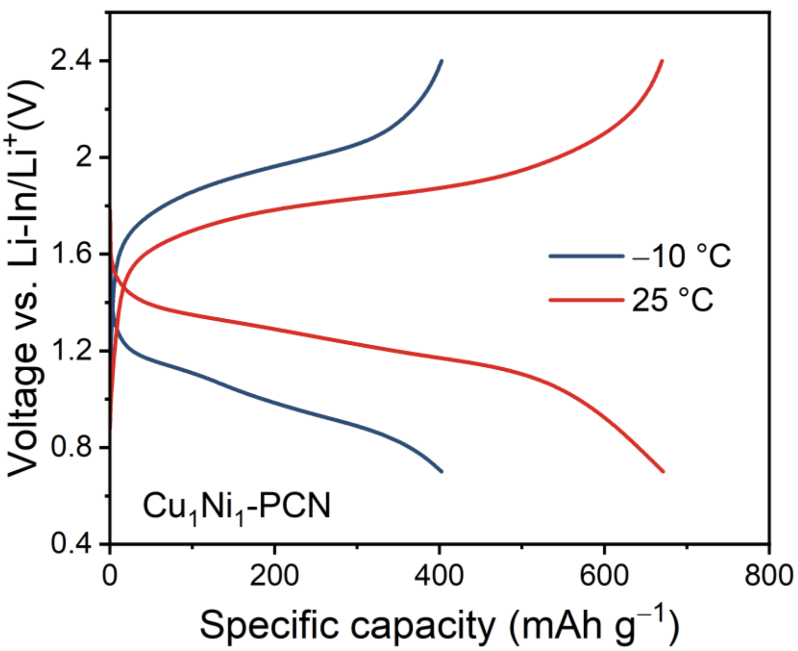

Figure S26 | Galvanostatic discharge-charge profiles of cells at different temperature.

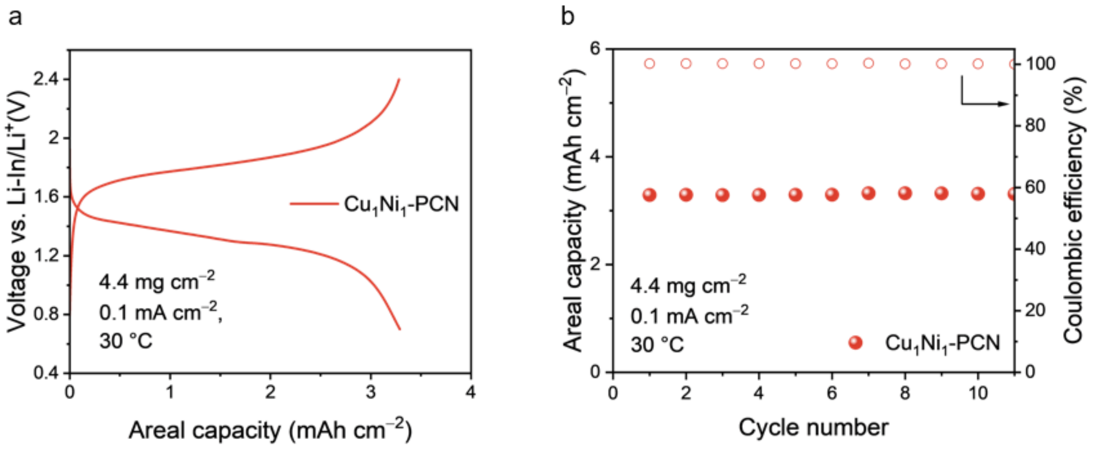

Figure S27 | a, Galvanostatic discharge-charge profiles of the ASSLSB with Cu_1Ni_1-PCN with a Li_2S loading of 4.4 mg cm^{-2} at 0.1 mA cm^{-2}. **b,** Cycling performance of the ASSLSB with Cu_1Ni_1-PCN with a Li_2S loading of 4.4 mg cm^{-2} at 0.1 mA cm^{-2}.

Table S1 | Comparison of metal content in different catalysts.

Catalysts	Metal content (wt%)	
	Cu	Ni
Cu_1-PCN	7.68	0
Ni_1-PCN	0	7.46
Cu_1Ni_1-PCN	7.92	7.55

Table S2 | EXAFS fitting results of the catalyst.

Sample	Shell	*N*	*R* (Å)	σ^2 (10^{-3}Å^2)	R factor
Cu_1-PCN	Cu-N	4.0 ± 0.4	1.95 ± 0.01	0.0065	0.011
Ni_1-PCN	Ni-N	4.0 ± 0.2	1.97 ± 0.02	0.0053	0.006
Cu_1Ni_1-PCN	Cu-N	1.9 ± 0.1	1.89 ± 0.01	0.0052	0.001
	Ni-N	3.9 ± 0.4	2.08 ± 0.25	0.0074	0.001
Cu foil	Cu-Cu	12.0*	2.54 ± 0.004	0.0081	0.002
Ni foil	Ni-Ni	12.0*	2.548 ± 0.002	0.0058	0.001

N, coordination number; *R*, distance between absorbing and backscattering atoms; σ^2, Debye-Waller factor to account for thermal and structural disorders; R factor as a measure of the goodness of fit.

Table S3 | Adsorption energies of sulfur species after adsorbing on different catalysts.

Sulfur species	Cu_1-PCN	Ni_1-PCN	Cu_1Ni_1-PCN
	Adsorption energies (eV)		
S_8	-1.28	-0.85	-0.99
Li_2S_2	-2.01	-1.38	-1.56
Li_2S	-3.24	-2.28	-2.55

Table S4 | Electrochemical performance comparison of recently reported ASSLSBs.

Cathode	Electrolyte	Anode	Current (C)	Cycle number	Capacity fading (%)	Temp	Ref.
S-Mo_6S_8	LGPS	Li-In	0.04	120	0.2%	70 °C	[1]
S@CNTs	LGPS	$Li_{0.8}Al$	0.05	100	0.132%	30 °C	[2]
S-super P	LPS	Li-In	0.5	100	0.155%	30 °C	[3]
S-CNT-LiI	LGPS	Li-In	1.2	1500	0.0086%	25 °C	[4]
$Li_2S/LiVS_2$	LPSC	Li-In	1	1000	0.023%	25 °C	[5]
$S_{9.3}I$	LPSC	Li-SP	0.1	400	0.034%	25 °C	[6]
S@LPS@A-PPy@NCNT	LPSC	Li-In	0.1	300	0.067%	30 °C	[7]
S-VGCF	LiSnPS	Li	0.1	200	0.02%	25 °C	[8]
S-KB-LPS	LPS	Li-In	0.1	100	0.129%	60 °C	[9]
S-LPSC-CNT	LPSC-CELs	Li-In	0.1	100	0.16%	25 °C	[10]
S-EVA@LGPS@SP	LGPS	Li-In	0.5	200	0.18%	30 °C	[11]
S-C-MIEC20	LPS	Li-In	2	1000	0.0024%	60 °C	[12]
Co@AB/S	LPSC	Li-In	0.5	1000	0.0217%	30 °C	[13]
CuS-Li_2S	LPSC	Li-In	1	500	0.055%	30 °C	[14]
Cu_1Ni_1-PCN-Li_2S	LPSC	Li-In	2<br>1	4500<br>1800	0%<br>0%	25 °C	This Work

Supplementary References

1. Li M, Liu T, Shi Z *et al.* Dense all-electrochem-active electrodes for all-solid-state lithium batteries. *Adv Mater* 2021; **33**: 2008723.
2. Pan H, Zhang M, Cheng Z *et al.* Carbon-free and binder-free Li-Al alloy anode enabling an all-solid-state Li-S battery with high energy and stability. *Sci Adv* 2022; **8**: eabn4372.
3. Zhu X, Jiang W, Zhao S *et al.* Exploring the concordant solid-state electrolytes for all-solid-state lithium-sulfur batteries. *Nano Energy* 2022; **96**: 107093.
4. Kim J T, Rao A, Nie H Y *et al.* Manipulating Li_2S_2/Li_2S mixed discharge products of all-solid-state lithium sulfur batteries for improved cycle life. *Nat Commun* 2023; **14**: 6404.
5. Kwok C Y, Xu S, Kochetkov I *et al.* High-performance all-solid-state Li_2S batteries using an interfacial redox mediator. *Energy Environ Sci* 2023; **16**: 610–8.
6. Zhou J, Holekevi Chandrappa M L, Tan S *et al.* Healable and conductive sulfur iodide for solid-state Li-S batteries. *Nature* 2024; **627**: 301-5.
7. Ma R, Fan Y, Jin Y *et al.* Construction of elastic and conductive channels for high-rate and high-areal-capacity sulfur cathodes in all-solid-state lithium-sulfur batteries. *Adv Energy Mater* 2024; **14**: 2304412.
8. Jiang Z, Peng H, Liu Y *et al.* A versatile $Li_{6.5}In_{0.25}P_{0.75}S_5I$ sulfide electrolyte triggered by ultimate-energy mechanical alloying for all-solid-state lithium metal batteries. *Adv Energy Mater* 2021; **11**: 2101521.
9. Alzahrani A S, Otaki M, Wang D *et al.* Confining sulfur in porous carbon by vapor deposition to achieve high-performance cathode for all-solid-state lithium–sulfur batteries. *ACS Energy Lett* 2021; **6**: 413–8.
10. Zhu G L, Zhao C Z, Peng H J *et al.* A self-limited free-standing sulfide electrolyte thin film for all-solid-state lithium metal batteries. *Adv Funct Mater* 2021; **31**: 2101985.
11. Zhu X, Jiang W, Wang L *et al.* Constructing resilient cross-linked network toward stable all-solid-state lithium-sulfur batteries. *Adv Energy Mater* 2024; **14**: 2304244.
12. Wang D, Gwalani B, Wierzbicki D *et al.* Overcoming the conversion reaction limitation at three-phase interfaces using mixed conductors towards energy-dense solid-state Li–S batteries. *Nat Mater* 2025; **24**: 243-51.
13. Zhong H, Su Y, Wu Y *et al.* Long-life and high-loading all-solid-state Li-S batteries enabled by acetylene black with dispersed Co-N_4 as single atom catalyst. *Adv Energy Mater* 2023; **13**: 2300767.
14. Yu Y, Singh B, Yu Z *et al.* A nanocrystallite CuS/nitrogen-doped carbon host improves redox kinetics in all-solid-state Li_2S batteries. *Adv Energy Mater* 2024; **14**: 2400845.
